# Development and Experimental Validation of Regularized Machine Learning Models Detecting New, Structurally Distinct Activators of PXR

**DOI:** 10.3390/cells11081253

**Published:** 2022-04-07

**Authors:** Steffen Hirte, Oliver Burk, Ammar Tahir, Matthias Schwab, Björn Windshügel, Johannes Kirchmair

**Affiliations:** 1 Division of Pharmaceutical Chemistry, Department of Pharmaceutical Sciences, Faculty of Life Sciences, University of Vienna, 1090 Vienna, Austria; steffen.hirte@univie.ac.at; 2 Dr. Margarete Fischer-Bosch-Institute of Clinical Pharmacology, University of Tübingen, 70376 Stuttgart, Germany; oliver.burk@ikp-stuttgart.de (O.B.); matthias.schwab@med.uni-tuebingen.de (M.S.); 3 Division of Pharmacognosy, Department of Pharmaceutical Sciences, Faculty of Life Sciences, University of Vienna, 1090 Vienna, Austria; ammar.tahir@univie.ac.at; 4 Departments of Clinical Pharmacology and Biochemistry and Pharmacy, University of Tuebingen, 72074 Tübingen, Germany; 5 Cluster of Excellence IFIT (EXC 2180) “Image-Guided and Functionally Instructed Tumor Therapies”, University of Tübingen, 72074 Tübingen, Germany; 6 Fraunhofer Institute for Translational Medicine and Pharmacology ITMP, Discovery Research Screening Port, 22525 Hamburg, Germany; bjoern.windshuegel@itmp.fraunhofer.de; 7 Department of Life Sciences and Chemistry, Jacobs University Bremen, 28759 Bremen, Germany

**Keywords:** pregnane X receptor, activators, machine learning, regularization, virtual screening

## Abstract

The pregnane X receptor (PXR) regulates the metabolism of many xenobiotic and endobiotic substances. In consequence, PXR decreases the efficacy of many small-molecule drugs and induces drug-drug interactions. The prediction of PXR activators with theoretical approaches such as machine learning (ML) proves challenging due to the ligand promiscuity of PXR, which is related to its large and flexible binding pocket. In this work we demonstrate, by the example of random forest models and support vector machines, that classifiers generated following classical training procedures often fail to predict PXR activity for compounds that are dissimilar from those in the training set. We present a novel regularization technique that penalizes the gap between a model’s training and validation performance. On a challenging test set, this technique led to improvements in Matthew correlation coefficients (MCCs) by up to 0.21. Using these regularized ML models, we selected 31 compounds that are structurally distinct from known PXR ligands for experimental validation. Twelve of them were confirmed as active in the cellular PXR ligand-binding domain assembly assay and more hits were identified during follow-up studies. Comprehensive analysis of key features of PXR biology conducted for three representative hits confirmed their ability to activate the PXR.

## 1. Introduction

The pregnane X receptor (PXR, NR1I2) is a key regulator in the defense against xenobiotic substances. The receptor transcriptionally regulates the expression of drug-metabolizing enzymes and transporter proteins [1]. Target genes of PXR include cytochrome P450 enzymes such as *CYP3A4*, as well as aldehyde dehydrogenases, multidrug resistance efflux pumps, glutathione S-transferases, sulfotransferases, and transporters such as ATP binding cassette efflux transporters [2,3]. Because of the mostly hydrophobic binding pocket of PXR, a broad variety of substances bind to, and activate, the receptor. As well as pregnanes, PXR is activated by bile acids [4], androstanes [5], cholesterol and its metabolites [6], colupulone [7], and endocrine disruptors [8], as well as various pesticides [9,10,11]. Moreover, PXR enables the metabolization of a large fraction of available prescription and herbal drugs [1].

PXR offers hepatoprotection by preventing the toxic accumulation of potentially harmful substances [12]. As a result, PXR decreases the severity of inflammatory bowel diseases like ulcerative colitis and Crohn’s disease [13]. The receptor also displays neuroprotective behavior by delaying the neurodegeneration in diseases such as Niemann-Pick type C1 [14]. However, PXR is also responsible for reducing the efficacy of drugs [15]: Drugs that are cleared through CYP3A4 promote their own metabolism by activating PXR—a process known as auto-induction [16]. More importantly, a drug can also induce the metabolism of co-administered drugs, reducing their efficacy significantly [1]. Examples of PXR activators include dexamethasone [17], paclitaxel [18], ciglitazone [19], troglitazone [19], rifampicin [20], ritonavir [21], avasimibe [22], clotrimazole [21], and, most prominently, hyperforin [23]. Hyperforin, which is the bioactive part of St. John’s wort and a potent activator of PXR [23], reduces the efficacy of the HIV protease inhibitor indinavir [24] and the anticancer therapeutic irinotecan [25].

In order to prevent drug–drug interactions it is important to understand which small-molecules activate PXR. However, the experimental testing of compounds for PXR activation is time consuming and expensive. In silico methods represent an attractive option to predict whether a given molecule is a PXR activator. The main challenge in the computational prediction of PXR activation lies in the correct representation of the receptor’s directed promiscuity, i.e., the property of binding a diverse but precise collection of substances [26].

A variety of different computational methods for the identification of PXR activators have been introduced so far [27]. For example, pharmacophore models generated from known ligands have been shown to be able to reflect the hydrophobic nature of the receptor’s ligand-binding pocket (LBP) as well as the hydrogen bond acceptor feature present in many activators [28,29,30,31,32,33,34]. Molecular docking has also been used for prediction of the binding of small molecules to PXR [16,31,32,34,35,36,37]. However, there are multiple reports of poor correlation between the docking score and biological activity [3,38]. Even the augmentation of docking with machine learning (ML) was reported to not improve the classification performance substantially [39]. QSAR methods including multiple-linear regression [40], partial least squares regression [41,42,43], regression trees [44,45], and polynomial neural networks [46] have been applied to predict the PXR activity of a substance. Higher-order QSAR methods like CoMFA have also been explored [35,46] but they generally suffer from similar problems to pharmacophore models because of the challenges involved in obtaining biologically meaningful structural alignments.

ML models encompass the majority of recent approaches for the prediction of PXR activation. Recursive partitioning [3,47,48], k-NN [40,47,49,50], Bayesian inference [37,51,52], probabilistic neural networks [3,39,49], artificial neural networks [50], support vector machines (SVMs) [3,47,49,50], decision trees [44], and random forests (RFs) [3,47] have been applied. On small data sets, SVMs and RFs proved to be effective [3,49]. When more training data are available, naive Bayes is also applicable [47,51,52]. A typical problem of ML models is their dependence on the training set. Especially for a complicated activity landscape as observed in PXR, ML models are prone to overfitting on the training set. As a result, such models typically pick structurally related compounds during virtual screening.

The goal of this work is the development of a machine learning approach that is able to identify activators of the human PXR even when they are structurally distinct from any of the PXR activators present in the training sets. To this end, we compiled a large data set of experimentally confirmed activators and non-activators of human PXR from public data sources on which we trained a number of ML models. A special feature of the newly devised model development and validation process is a new scoring function that promotes the generalization power of models over their performance in commonly applied cross-validation tests. The result of this modeling strategy are models that are able to predict PXR activation even for compounds that are structurally dissimilar from those represented in the training data, as we show in a large-scale virtual screening study of more than 7 million compounds and subsequent biological testing of selected substances. The computational approach can be employed, for example, in the optimization of screening libraries, the (de-) prioritization of hits, and for compound optimization.

## 2. Materials and Methods

### 2.1. Data Sets

PubChem PXR data set: PubChem Bioassay AID 720659 (“qHTS assay for small molecule activators of the human pregnane X receptor (PXR) signaling pathway”) was downloaded from the PubChem BioAssay database [53,54] via the PUG REST interface. Any entries lacking a compound ID (CID) or having the activity label “inconclusive” were discarded. Subsequently, the SMILES representations of the remaining compounds were retrieved via the PUG REST interface using the CIDs as queries.

ToxCast PXR data set. The ToxCast & Tox21 database (“InvitroDB”) summary files were retrieved from the web site of the United States Environmental Protection Agency [55]. The archive provides activation data for PXR in four different Attagene assays: ATG_PXRE_CIS_up, ATG_PXRE_CIS_dn, ATG_PXR_TRANS_up, and ATG_PXR_TRANS_dn. The keywords “CIS” and “TRANS” indicate the mode of activation whereas “up” and “dn” denote the direction of assay measurement. Since PXR has low basal activity, we excluded the “dn” assays. The remaining two assays, ATG_PXRE_CIS_up and ATG_PXR_TRANS_up, were used to infer a binary activation label for each substance. For the purpose of this study, a compound was defined to be an activator (non-activator) if it has a hitc value of one (zero) in both assays. Compounds with disagreeing hitc values in both assays were considered inconclusive and removed. Using the chemical summary file from the ToxCast archive, compounds were linked to the registration number in the Chemical Abstract Service (CAS). Based on the CAS registration number, the respective InChIs were obtained with the NCI/CADD Chemical Identifier Resolver [56].

Literature PXR data set: Matter et al. [44] have compiled a set of 434 small molecules with binary PXR activation data from the literature. These data were obtained directly from the authors in SD file format.

Reference data sets of drugs, cosmetics, and pesticides. The complete database of approved, experimental, and withdrawn drugs was obtained from the DrugBank web site [57,58]. The COSMOS DB cosmetics database (COSMOSDB) and the pesticide chemical search database (EPAPCS) were retrieved from the CompTox dashboard [59,60,61].

Compound library for virtual screening: The subset of in-stock compounds of the MolPort compound library was obtained from the vendor’s website [62].

### 2.2. Data Preprocessing

Unless stated otherwise, all data sets were processed separately according to the following protocol: After removal of the minor components (e.g., salts), the structures were standardized with the methods fragment_parent and tautomer_parent of the MolVS Python module [63]. Molecules with molecular weight below 200 Da and molecules containing an element other than H, B, C, N, O, Si, P, S, Se, F, Cl, Br, or I were discarded (these filtering steps were not applied to the compounds from the reference data sets). Duplicate molecules were identified by generating and comparing InChIs with RDKit [64]. For a set of molecules with identical InChIs, one instance was kept if the activity values were identical; otherwise, all instances were removed. Table 1 shows the number of compounds included in the raw and the preprocessed data sets, and the number of compounds removed during the individual steps.

### 2.3. Feature Calculation

For each molecule of the PubChem, ToxCast, and literature PXR data set, 17 physicochemical descriptors were computed with RDKit: number of heavy atoms, oxygen atoms, nitrogen atoms, sulfur atoms, hydrogen bond acceptors, hydrogen bond donors, rings, rotatable bonds, halogens, sp3 hybridized carbons, and aromatic atoms, topological polar surface area (TPSA), molecular weight, refractivity, logP, estimated solubility, and fraction of rotatable bonds.

### 2.4. Experimental Approaches

#### 2.4.1. Chemicals and Reagents

Rifampin was provided by Merck Chemicals (Darmstadt, Germany); DMSO and 1α,25-dihydroxyvitamin D3 were purchased from Sigma-Aldrich (Munich, Germany); CITCO and SPA70 were obtained from ENZO Life Sciences (Lörrach, Germany) and Axon Medchem (Groningen, The Netherlands), respectively. Minimum essential medium (MEM), William’s E medium and Trypsin-EDTA solution were purchased from Thermo Fisher Scientific (Waltham, MA, USA). L-glutamine, non-essential amino acids, sodium pyruvate and penicillin-streptomycin mixture were provided by Biozym (Hessisch Oldendorf, Germany). Fetal bovine serum (FBS) was obtained from Biowest (Nuaillé, France).

#### 2.4.2. Compound Purity Checks

The three compounds selected for comprehensive biological characterization were subjected to purity checks using a UHPLC-DAD-CAD-MS system -UHPLC (ultra-high pressure liquid chromatography) coupled to three detectors: (1) UV-DAD (diode array detector) (2) CAD (corona aerosol detector) (3) MS (mass spectrometer). The Ultimate 3000 UHPLC-DAD--CAD system (Thermo Fisher Scientific, San Jose, CA, USA) was equipped with a reversed-phase C18 column (Kinetex, Torrance, California, CA, USA; 2.1 mm × 15 cm, 2.6 μm, C18 100 Å). Mobile phase A (H2O/FA, 100:0.01) and mobile phase B (ACN) were degassed prior to their usage. A 20 min binary gradient with flow rate set to 350 μL/min was applied as follows: 0–1 min, 5% mobile phase B; 2–12 min, 5–95% mobile phase B; 13–17 min, 95% mobile phase B; 18–20 min re-equilibration with 5% mobile phase B. Five microliters of each compound (DMSO stock solutions diluted 1:100 in MeOH) were injected followed by a blank injection to ensure proper column washing and equilibration. DAD and CAD detection provided the chromatograms used to assess the purity of the compounds. Mass spectrometric detection, to confirm the identity of the compounds, was performed with an LTQ-XL linear ion trap mass spectrometer (Thermo Fisher Scientific) using the HESI source (300 °C heater temperature, 40/10/1 arb. units for the sheath, aux and sweep gasses respectively and 3.5 Kv spray voltage at 275 °C capillary temperature) to achieve negative/positive ion mode ionization. MS scans were performed with an m/z range from 150 to 2000. MS/MS scans of the 3 most abundant ions were achieved through collision-induced dissociation (CID) fragmentation at 30% normalized collision energy.

#### 2.4.3. Cell Culture

HepG2 (HB-8065, ATCC, Manassas, VA, USA) and H-P cells (HepG2 cell clone stably overexpressing human PXR) [65] were cultivated in MEM, supplemented with 10% FBS, 2 mM L-glutamine, 100 U/mL penicillin, and 100 µg/mL streptomycin. In drug treatments, dextran-coated charcoal-stripped FBS replaced regular FBS.

HepaRG cells (Biopredic, Rennes, France) were cultivated in phenol red-free William’s E medium, supplemented with 10% FBS, 2 mM glutamine, 100 U/mL penicillin, 100 µg/mL streptomycin, 5 µg/mL insulin, and 50 µM hydrocortisone. For chemical treatment, performed in technical triplicates, 1.0 × 10^5^ cells were seeded per well of a 12-well plate. At confluence, the growth medium was supplemented with 2% DMSO and cells were cultivated for a further 2 weeks to differentiate them into hepatocytes [66]. Then, cells were adapted for 48 h to induction medium (growth medium with only 2% FBS and 0.2% DMSO). Chemical treatment was started for another 48 h, with daily medium change. Experiments were conducted 3 times independently.

Cells were routinely tested by PCR for mycoplasma contamination using the VenorGeM Classic kit (Minerva Biolabs, Berlin, Germany).

#### 2.4.4. Cell Viability

HepG2 or H-P cells were seeded into white flat-bottom CELLSTAR^®^ 96-well plates with µClear^®^ bottom (Greiner Bio-One, Frickenhausen, Germany), with 4.0 × 10^4^ cells per well in a volume of 100 µL. The next day, cells were treated with compounds, ranging from 1 to 50 µM, or vehicle only (0.1–0.17% DMSO). Each treatment was performed in technical triplicates. After 24 h of treatment, cell viability was determined using the CellTiter-Glo^®^ luminescent cell viability assay (Promega, Madison, WI, USA), as specified by the manufacturer. Luminescence was measured with the 2300 EnSpire multimode plate reader (Perkin Elmer, Rodgau, Germany). Experiments were conducted 3 times independently.

#### 2.4.5. Plasmid Constructs

Expression plasmids encoding human nuclear receptors CAR1 and CAR3 [67] and RXRα [68] and VDR [69] have all been described previously.

Expression plasmids encoding fusion proteins of GAL4-DNA binding domain (DBD) and the receptor interaction domains (RID) of nuclear receptor co-activator (NCOA) 1 (residues 583-783) [70], nuclear receptor co-repressor (NCOR) 2 (residues 1109-1330), or PXR ligand binding domain (LBD) helix 1 part (residues 132-188), as well as expression plasmids encoding fusion proteins of the VP16 activation domain (AD) and the whole (residues 108-434) or part (189-434) of the PXR-LBD [69] have been described earlier.

The following firefly luciferase reporter gene plasmids were used: *CYP3A4* enhancer/promoter reporter gene plasmid pGL4-CYP3A4(7830Δ7208-364) [71]; *CYP2B6* enhancer/promoter reporter gene plasmid pB-1.6k/PB/XREM [72]; pGL3(DR3)_3_Tk, with a trimer of *CYP3A23* direct repeat (DR) 3 motif [73]; GAL4-dependent pGL3-G5 [70].

For normalization of transfections, Renilla luciferase expression plasmid pGL4.75[hRLuc/CMV] (Promega) was used.

#### 2.4.6. Transient Transfections, Promoter Reporter Gene, and Mammalian Two-Hybrid Assays

Transient transfections of HepG2 and H-P cells were set up according to the batch protocol for jetPEI^®^ (Polyplus, Illkirch, France), as described previously [74]. The following plasmid amounts (per well of 96-well plate) were used:

For mammalian two-hybrid PXR LBD assembly assay, 0.24 µg pGL3-G5, 0.03 µg each of expression plasmids encoding GAL4-DBD/PXR(132-188) and VP16-AD/PXR(189-434) fusion proteins; for mammalian two-hybrid co-factor interaction assays, 0.225 µg or 0.24 µg pGL3-G5, 0.03 µg expression plasmid encoding VP16-AD/PXR-LBD(108-434) fusion and 0.03 µg expression plasmids encoding GAL4 DBD/NCOR2-RID or GAL4 DBD/NCOA1-RID fusions. Additionally, 0.015 µg of RXRα expression plasmid was added in NCOR2 co-repressor interaction; in CYP3A4 reporter gene assay conducted in H-P cells: 0.3 µg pGL4-CYP3A4(-7830Δ7208-364); in nuclear receptor selectivity assays: 0.26 µg/0.23 µg pB-1.6k/PB/XREM (as reporter for CAR1/CAR3) or pGL3(DR3)3Tk (as reporter for VDR) and 0.03 µg either CAR1, CAR3, or VDR expression plasmids. In addition, 0.03 µg RXRα expression plasmid was added to CAR3 transfections. For all assays, 0.01 µg of Renilla luciferase expression plasmid pGL4.75[hRLuc/CMV] was added to allow for normalization.

24 h after transfection, cells were treated with chemicals. After a further 24 h, cells were lysed, and Firefly and Renilla luciferase assays were performed as described previously [74]. All transfections were done independently for 3–5 times, each in technical triplicates, and with at least two different preparations of plasmids.

#### 2.4.7. RNA Preparation and Reverse Transcription Quantitative Real-Time PCR Analysis

The NucleoSpin RNA kit (Machery-Nagel, Düren, Germany) was used to prepare total RNA from chemically treated differentiated HepaRG cells. Integrity of the isolated RNA was analyzed by formaldehyde-agarose gel electrophoresis. cDNA was synthesized as described previously [75].

Relative quantification analyses (ΔΔCt) were conducted in technical triplicates with TaqMan RT-PCR using the BioMark HD system and Flex Six Gene Expression Integrated Fluidic Circuits (Fluidigm, South San Francisco, CA, USA), as described previously [75]. TaqMan gene expression assays were either the commercial predesigned assays Hs00184500_m1 (ABCB1) and Hs00604506_m1 (CYP3A4) (Thermo Fischer Scientific) or, in the case of CYP2B6, have been described earlier [69]. Data were analyzed as described before [75] and gene expression levels were normalized to corresponding 18S rRNA levels, as determined using the 18S rRNA assay previously described [76].

## 3. Results and Discussion

### 3.1. Analysis of the Data Available for Model Development

**Figure 1:**
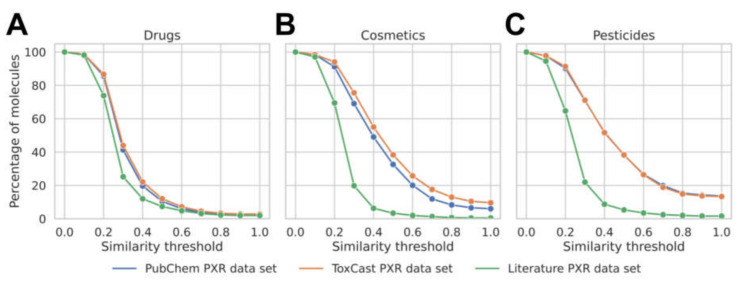
Fraction of molecules (*y*-axis; (**A**) drugs, (**B**) cosmetics, (**C**) pesticides) in each reference data set covered by the PubChem, ToxCast, and Literature PXR data sets (indicated by color) at different similarity thresholds (*x*-axis).

For the purpose of model development and testing, sets of compounds with measured PXR activation data were retrieved from the PubChem Bioassay database, the ToxCast database, and the literature (Table 1; see Methods for details). In order to understand how well the individual PXR data sets represent the chemical space relevant to biomedical research, we compared them with established reference data sets of approved, withdrawn, and experimental drugs (DrugBank) and a collection of cosmetics and pesticides (CompTox dashboard). As shown in Figure 1, at a similarity threshold of 0.7 (calculated as the Tanimoto coefficient of the corresponding Morgan fingerprints with a radius of 2 and a length of 2048 bits), which indicates that two molecules are in a close structural relationship, the PubChem PXR data set covers 4% of the approved drugs data set, 12% of the cosmetics data set, and 20% of the pesticides data set. The ToxCast PXR data set shows similar coverage to the PubChem PXR data set, whereas coverage is lower for the literature PXR data set (which is expected, given its smaller size).

In terms of molecular scaffolds (described as Murcko frameworks), the PXR data sets cover in the range of 3 to 4% of the scaffolds observed in the approved drug set, 5 to 22% of the scaffolds observed in cosmetics, and 7 to 25% of scaffolds observed in pesticides (Table 2). It is noteworthy that, despite its smaller size, the literature PXR data set covers approximately the same portion of the scaffolds observed in drugs as the other data sets. However, the PubChem and ToxCast PXR data sets have coverage that is more than three times higher that of the scaffolds observed in cosmetics and pesticides than the literature PXR data set. With Murcko scaffolds representing, on average, just ~1.6 molecules, the literature PXR data set is clearly more diverse than the PubChem and ToxCast data sets, in which Murcko scaffolds represent, on average, ~2.5 molecules each.

**Figure 2:**
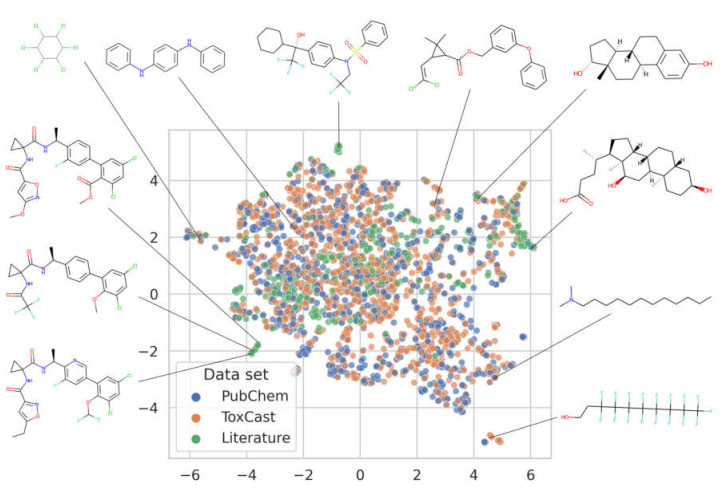
2D embedding of the molecules contained in the PubChem, ToxCast, and literature PXR data sets. For almost every molecule (99%), all of its neighbors that have a Tanimoto similarity (based on Morgan fingerprints with a radius of 2 and a length of 2048 bits) greater than 0.7 are within a distance of 0.4 in the embedding.

In order to obtain a better understanding of the molecular diversity of the individual PXR data sets we performed a dimensionality reduction and projection onto a 2D surface with Uniform Manifold Approximation and Projection (UMAP, n_epochs = 50,000, n_neighbors = 40, min_dist = 0.3, metric = “jaccard”) [77]. UMAP largely preserves local structure, meaning, in the specific context, that similar molecules are placed in proximity to each other on the 2D surface. The distribution of the points shown in Figure 2 indicates that the PubChem PXR data set and the ToxCast PXR data set are very similar on a global scale. In contrast, the literature PXR data set is characterized by multiple analogue series (visible as clusters of data points) that do not have structurally related compounds in the ToxCast and PubChem PXR data sets.

Based on these observations, we selected the PubChem PXR data set as the training set for machine learning. Not only does this data set cover the chemical space spanned by all data sets but it also is the largest consistent PXR data set available in the public domain (note that the ToxCast database contains more PXR bioactivity data points, but they originate from two different assays). Henceforth, we refer to the PubChem PXR data set as the training set for ML. The ToxCast and the literature PXR data sets served as test sets. From these test sets we removed any compounds present in the training set (based on InChI representations). This reduced the ToxCast PXR data set by 393 compounds down to 768 compounds and the literature PXR data set by 39 compounds down to 370.

### 3.2. Model Development and Internal Validation

A total of six binary models for the prediction of PXR activators and non-activators were optimized and trained on the PubChem PXR data set. These models result from the combination of two ML algorithms (RF, SVMs) with three sets of molecular descriptors (physicochemical descriptors (PCs), fingerprints (FPs), and the combination of both). The hyperparameters of the individual models (see Table S1) were optimized during a grid search within a 5-fold cross-validation framework maximizing the average Matthews correlation coefficient (MCC) on all validation folds, i.e.,

(1) $$validation\;performance=\frac{1}{k}\sum_{i=i}^{k}MCC_{val}^{\left(i\right)},$$

where *k* = 5 is the number of folds in the cross-validation, and $MCC_{val}^{\left(i\right)}$ is the model’s MCC on the i-th validation fold.

**Figure 3:**
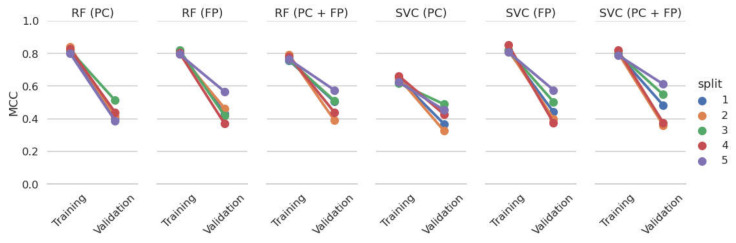
The MCC on the training and validation set of each ML model (denoted by the sub chart titles) and cross-validation split (indicated by color).

During cross-validation we observed a high discrepancy between training and validation performance for all models. Figure 3 shows that each of the classifiers performed worse on the validation fold compared to the training folds for each cross-validation split. This is an indication that the models are likely overfitting on the training examples. Since the training and validation examples originate from the same data set, cross-validation can be seen as a moderately challenging testing scenario. The performance of these models will decrease even further if they are tested on molecules that differ substantially from the compounds in the PubChem PXR data set.

Our aim was to develop models that perform well also on unseen data and hence are able to identify PXR activators that are structurally distinct to those represented by the training data. In order to identify the best model, we devised a scoring function that takes both CV performance and the generalization capabilities of a model into account:

(2) $$gap\;penalized\;performance=\frac{1}{k}\sum_{i=i}^{k}MCC_{val}^{\left(i\right)}-\frac{1}{k}\sum_{i=i}^{k}\left|MCC_{train}^{\left(i\right)}-MCC_{val}^{\left(i\right)}\right|,$$

where $MCC_{train}^{\left(i\right)}$ is the model’s MCC on the i-th training fold. Apart from the average validation score (expressed as $MCC_{val}^{\left(i\right)}$ averaged over the CV-folds), the function uses the average train-validation gap (expressed as the difference between $MCC_{train}^{\left(i\right)}$ and the MCC obtained on the validation fold, $MCC_{val}^{\left(i\right)}$) as a penalty term. In this setup, a model with a large discrepancy between training and validation score can be outperformed by a weaker model with an agreement in training and validation performance.

The ToxCast and literature PXR data sets serve as two different test sets, each demonstrating a distinct setup. As described above, the ToxCast and PubChem PXR data sets show a highly similar distribution of compounds and scaffolds. This indicates that these two data sets fulfill the iid condition that is sufficient for an ML model to successfully learn a structure-activity-relationship on the training set that can be transferred to the test set. However, the literature PXR data set differs from the PubChem PXR data set to a degree that both data sets can be assumed to originate from different generating distributions.

For each of the six types of classifiers, we selected the model that maximizes the new scoring function in the hyperparameter search (see optimal parameters in Tables S2 and S3). As the new scoring scheme has analogies with regularization techniques, the models obtained by optimizing the new score are called regularized models.

Together with the six models from the previous hyperparameter optimization, we tested all twelve models on the ToxCast and literature PXR data sets. The cross-validation as well as results obtained on the test sets are reported in Table 3 for all models.

The models were optimized on the MCC score. MCCs reached from 0.41 to 0.48 in the cross-validation scenario. Including the train-validation gap in the optimization score resulted in models achieving an MCC ranging from 0.31 to 0.43, never exceeding the cross-validation performance of their counterpart models that were optimized on the pure MCC score. On the contrary, four out of six models recorded a drop in MCC and AUC value of at least 0.06 and 0.02 when switching to the score including the penalization term. However, the test MCC scores indicate that the models obtained from the new scoring function have an advantage on unseen data. The RF classifier built on all available features benefits most from the new optimization score, with an increase of 0.21 and 0.12 in MCC on the literature and ToxCast PXR data sets, respectively. On the other hand, the AUC values increase in only two of the six model templates when gap penalization is included.

**Figure 4:**
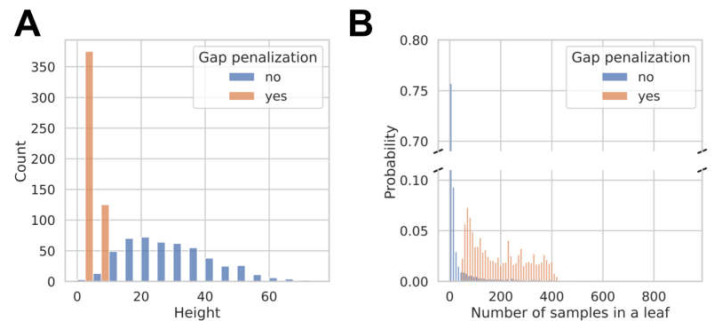
Comparison of an RF model optimized with (orange) or without (blue) a gap penalization term. (**A**) Number of decision trees (*y*-axis) having a certain height (*x*-axis); (**B**) Probability of the selection of a leaf in the RF (*y*-axis) containing a specific number of training samples (*x*-axis).

The regularized models differ fundamentally in their hyperparameters (see Table S2). For the RF models, the min samples split and the min samples leaf parameters were at least a factor of 4 times higher for the models selected based on the score including the gap penalty as compared to those selected based on the MCC. The min samples split and min samples leaf parameters affect the height of the resulting decision trees, and, in consequence, the number of training examples landing in the leaves of the decision trees and the agreement of the training labels. Using the RF model template with physicochemical and fingerprint features as an example, Figure 4 shows that its decision trees are deep (with a median height of 28) and have few samples per leaf (with a median of 31 samples per leaf) when optimized without gap penalization. Including the training-validation gap in the scoring function results in the selection of models with more shallow trees (with a median height of 4), containing more samples per leaf (median of 172). These models are more robust and capable of learning general rules of PXR activation instead of relying only on the nearest neighbors of a compound.

Likewise, in the case of SVMs, the models selected based on the score including the gap penalty have a C value that is at least a factor of 10 lower than that of the SVMs selected based on the MCC. The parameter C controls the regularization of the model. By definition, an SVM penalizes observations that are incorrectly classified by a factor of C. A large penalty term forces the model to find a perfect separation between PXR activators and non-activators. Consequently, decreasing C leads to models that allow errors on the training set in order to avoid overfitting. Overall, the observations for the RF and SVM models indicate that the addition of the train-validation gap enforces the selection of models that are less complex.

**Figure 5:**
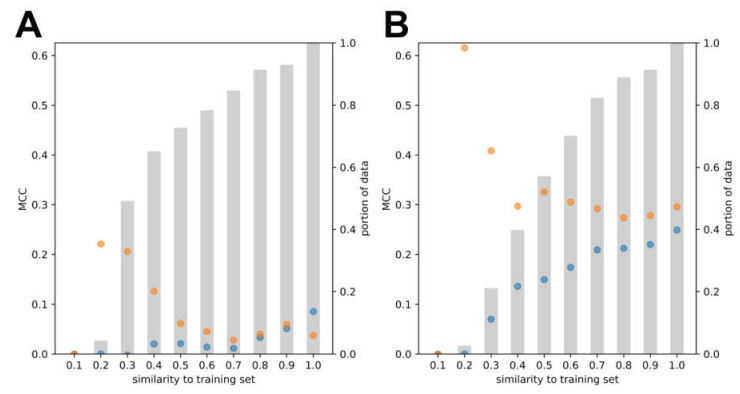
Performance of the regularized model (orange) and the baseline model (blue) as a function of the maximum similarity of the test molecules to the compounds in the training set, for (**A**) the ToxCast PXR data set and (**B**) the literature PXR data set. The percentage of compounds of the full test data set (computed as subset size/test set size) is visualized with gray bars.

The different model structures have an impact on the classification process. To demonstrate this, we evaluated the classifiers on different portions of the test data that vary in their similarity to the training set. For each molecule in the test set, we calculated the maximum similarity with respect to all molecules in the training set. Then, for each threshold t from 0.1 to 1.0 (in steps of 0.1) we created a subset St of the test set that includes all molecules with a maximum similarity of at most t. Using the RF model based on molecular fingerprints as an example, we computed the MCC score on all subsets of each test set as shown in Figure 5. Although both the baseline and regularized model have similar MCC on the full test sets at a similarity threshold of 1, the regularized model gains most of its performance on non-similar molecules while the baseline model is best at predicting similar molecules.

### 3.3. Analysis of Feature Importance

**Figure 6:**
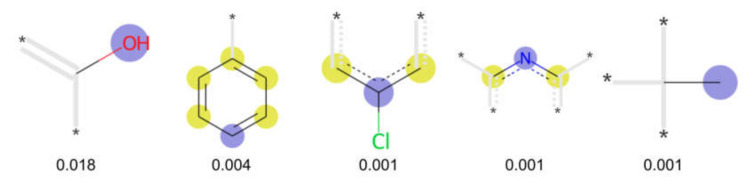
Examples of fingerprint features used by the regularized RF model. For each fingerprint bit, blue shades indicate the central atom, and yellow shades indicate aromatic environment atoms. The asterisk (*) denotes adjacent aliphatic or aromatic atoms. Feature importance values are noted below each substructure.

We investigated the impact of the different feature sets and individual features on model performance. As shown in Table 3, models trained on the combined set of physicochemical descriptors and molecular fingerprints do not perform substantially better than those trained on physicochemical descriptors only. The benefit in terms of MCC values did not exceed 0.04. This indicates that the 17 interpretable physicochemical features (such as molecular weight and TPSA; see Methods) already suffice for classification and adding the 8192 fingerprint features does not add much value. In order to provide a quantitative assessment, we computed the feature importances of both RF models that were trained on physicochemical and fingerprint features. For the model optimized with gap penalty, only 84 of the 8209 features have a positive feature importance value (Table S4), indicating that these are the only features the model uses for classification. All 17 physicochemical features are present in this list, leaving room for only 67 relevant fingerprint features (Figure 6). The three highest-ranked physicochemical features are esol, molecular refractivity, and logP with feature importance values greater than 0.1 (see Table S4). In contrast, the most relevant fingerprint features reach a feature importance value of at most 0.018.

### 3.4. Prospective Screening for PXR Activators

The in-stock collection of the MolPort database, containing 7,233,399 compounds, was screened for new potential PXR activators with the model that showed the best performance on unseen data (i.e., RF model trained on physicochemical descriptors and fingerprint optimized with gap penalization). We henceforth refer to this model as the guiding model. All five other models optimized with gap penalization were used to create a consortium of models supporting decision making.

The virtual screening resulted in a rank-ordered list of compounds. From the top-ranked positions of this list we selected a total of 31 compounds for purchasing and experimental validation (Table 4), taking the following aspects into account:

1. High confidence in predictions: Any selected compound must be predicted as a PXR activator by all of the three RF and three SVM models.
2. Novelty: The selected compounds must be structurally distinct to any known PXR ligands. This means that, at the time of selection, chemical structure similarity searches with CAS Scifinder did not result in the retrieval of any known, structurally related PXR ligands. More specifically, a minimum similarity threshold of 70 was used for the searches in CAS Scifinder, meaning that the platform would report literature even for rather distantly related PXR agonists.
3. Purchasability: The selected compounds must be available from MolPort in sufficient quantities (5 mg) and at moderate costs.

Among the 31 selected compounds are a cluster of 11 diphenylamines (“cluster A”) and a cluster of 10 chrysanthemic acid esters (“cluster B”). Five of the chrysanthemic acid esters are characterized by a 4-{[(5Z)-4-oxo-2-sulfanylidene-1,3-thiazolidin-5-ylidene]methyl}benzyl substituent.

#### Experimental Validation

The selected compounds were tested for their ability to bind to PXR using the cellular mammalian two-hybrid PXR ligand binding domain (LBD) assembly assay (Table 4), which can be regarded as a cellular equivalent of biochemical PXR ligand binding assays [71].

Two of the eleven compounds of the diphenylamine cluster (**9** and **11**) showed moderate activity in the PXR-LBD assembly assay, with 33% activation by both compounds. The other compounds of this cluster were inactive with activity of less than 10%). Compounds **9** and **11** are characterized by more bulky, hydrophobic alkyl or aryl substituents in ortho position of one of the benzene moieties.

Within the cluster of chrysanthemic acid esters, four compounds showed activity in the PXR-LBD assembly assay: **13** (28%) and **15** (26.5%), both characterized by nitrogen-rich, five-membered heteroaromatic rings, and 18 (40%) and 17 (45%), both characterized by a substituted 4-{[(5Z)-4-oxo-2-sulfanylidene-1,3-thiazolidin-5-ylidene]methyl}benzyl moiety. Thiazolidin-4-one-derivatives have been previously reported as potent agonists of the constitutive androstane receptor (CAR) [78].

Among the singleton (virtual) hits, **22** and **25** were identified as most promising in the PXR-LBD assembly assay, with activities of 137% and 91%, respectively.

Overall, twelve of the selected compounds showed at least weak activity (>10% activation in comparison to rifampicin). Compound **22** almost reached the activity level of rifampicin and **25** even exceeded the activity induced by rifampicin.

### 3.5. Hit Follow-Up and SAR Analysis

In order to further investigate the influence of molecular structure on compound activity in the PXR-LBD assembly assay, we purchased analogs of the most interesting compounds, **17**, **22** and **25**.

Among the compounds structurally related to **25**, the initial hit (**25**) and **34** exhibited the highest activities in the PXR-LBD assembly assay (Table 5). From the SAR analysis, we learn that the -CF3 moiety at R1 is likely beneficial but certainly not essential for activity. Subtle changes of the substituent in R3 can have profound effects on the bioactivity of the compound. For example, the replacement of the N,N-ethyl-2-methylallyl moiety in R3 (**25**) by a 4-phenyl-3,6-dihydropyridine moiety (**37**) led to the abolishment of the biological activity.

In contrast to the follow-up on **25**, in the case of **22**, the testing of analogs (all diphenylthiazolimines) resulted in the identification of three compounds (**42**, **43**, **44**) that were more potent than the initial hit (Table 6). From the SAR analysis, we can learn that the moiety in R3 is decisive for bioactivity; a small, hydrophobic moiety is preferred at this location.

The follow-up on the benzylidenethiazolidinone **17** also resulted in more potent hits, in particular **53** and **57** (Table 7). Neither **53** nor **57** carry the chrysanthemic ester moiety that is prominent among the initial hits. The bioactivity data indicate that R3 and R4 have a decisive impact on bioactivity (cp. **53** and **55**).

### 3.6. Characterization of Prototypical Compounds

Nuclear receptor LBD assembly assays do not discriminate between agonists and antagonists, which both result in LBD assembly [79]. In order to differentiate these two types of ligands we performed follow-up analyses with one prototypical, strong PXR-interacting compound of each analog series. For the follow-up study, we chose **25**, **42** and **53**. Compound **25** is the strongest tested effector of its kind. Compound **42** is the second-strongest effector of its class (we needed to discard the strongest effector of its class, **44**, as we found, during mass spectrometry analysis of the substance, that it did not meet the purity threshold of a minimum of 90%; mass spectrometry data for **25**, **42** and **53** is provided in Figure S1). Compound **53** is the second strongest effector of its class (also in this case, the strongest effector of its class, **57**, needed to be discarded due to purity issues).

**Figure 7:**
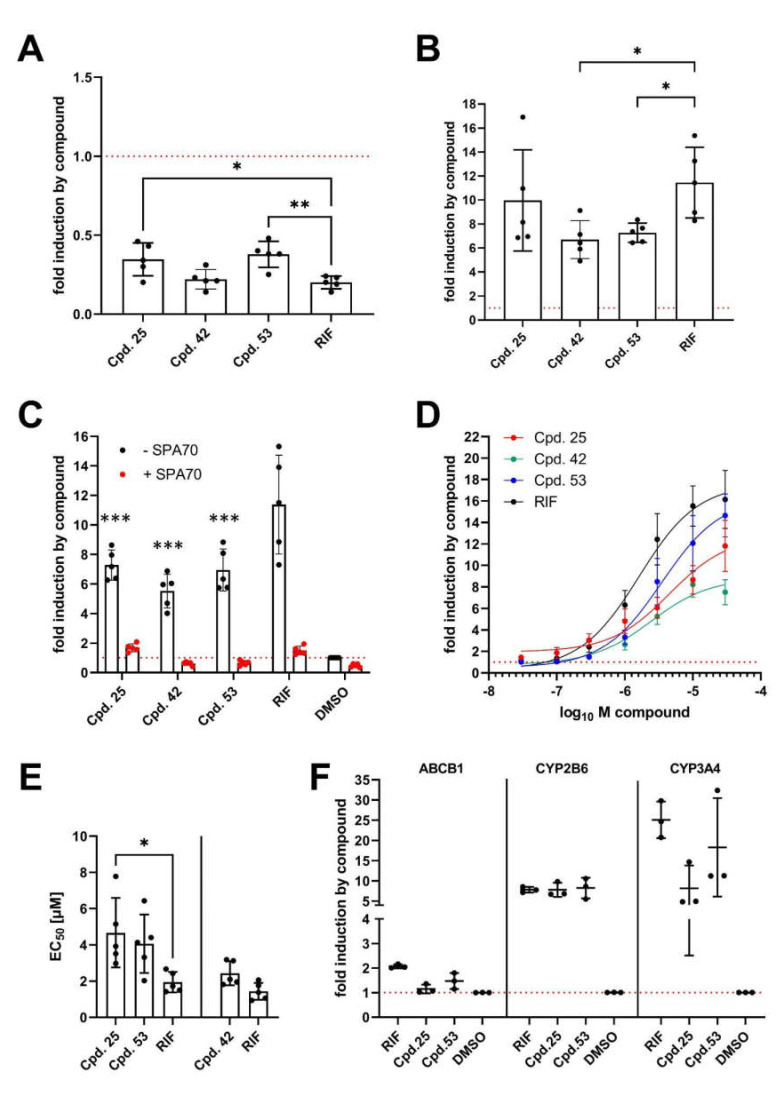
Characterization of novel PXR effectors by analysis of co-factor interaction assays, *CYP3A4* reporter gene activation and induction of endogenous PXR target genes. (**A,B**) HepG2 cells were co-transfected with expression plasmid encoding VP16-AD/PXR-LBD (108–434) fusion protein and expression plasmids encoding fusion proteins of (**A**) GAL4-DBD/NCOR2-RID or (**B**) GAL4- DBD/NCOA1-RID. Transfected cells were treated with 0.1% DMSO or 10 µM of the indicated compounds for 24 h. (**C**,**D**) H-P cells were transfected with the CYP3A4 promoter/enhancer reporter gene plasmid and treated for 24 h with (**C**) 0.1 % DMSO or 10 µM of the indicated compounds in the absence (-SPA70) or presence (+SPA70) of 5 µM SPA70 or (**D**) with increasing concentrations of the indicated compounds. The fold induction values of normalized luciferase reporter gene activity of co-transfected pGL3-G5 (**A**,**B**) or CYP3A4 promoter/enhancer (**C**,**D**) by chemical treatment, as compared to vehicle DMSO only (designated as 1 and indicated by red, dotted lines), are presented as scatter plots with means (columns) ± S.D. (*n* = 5) (**A**–**C**) or as nonlinear fit of concentration-dependent response with means ± S.D. (*n* > 5) (**D**). (**E**) shows scatter plots with means (columns) ± S.D. (*n* = 5) of EC50 values derived from the experiments shown in (**D**). (**F**) Differentiated HepaRG cells were treated with 0.1% DMSO or 10 µM of the indicated compounds for 48 h. mRNA expression of the indicated genes was determined by TaqMan RTqPCR and normalized to the expression of 18S rRNA. Data are presented in scatter plots with means and S.D. (*n* = 3). Expression was calculated as fold induction by chemical treatment. Differences to respective treatment with rifampicin were analyzed by repeated measures using one-way ANOVA (**A**,**B**,**E**) or repeated measures using two-way ANOVA (**C**) with Dunnett’s multiple comparisons test. * *p* < 0.05; ** *p* < 0.01; *** *p* < 0.001.

First, we analyzed the capacity of the selected compounds to modulate the interaction of PXR with co-factors, using mammalian two-hybrid assays. Similarly to rifampicin, all novel PXR effectors resulted in the release of co-repressor NCOR2 from PXR and, by that, demonstrated agonist activity (Figure 7A). While **42** was as efficient as rifampicin, **25** and **53** demonstrated reduced capacity to release the co-repressor. Figure 7B shows that the novel compounds resulted also in the recruitment of co-activator NCOA1, with 25 being as efficient as rifampicin, and **42** and **53** showing reduced recruitment. These results add further evidence that the compounds act as PXR agonists.

Secondly, as PXR agonists have to activate the transcriptional activity of the receptor, we analyzed activation of transiently transfected *CYP3A4* enhancer/promoter reporter gene by compound treatment in cells with stable PXR overexpression. Figure 7C shows that transactivation of the *CYP3A4* reporter by the novel compounds was weaker than by rifampicin. Dependency on PXR was demonstrated unequivocally by co-treatment with the specific PXR antagonist SPA70 [80], which completely blocked reporter activation in all cases. Concentration response analyses, using up to 30 µM of the compounds, further confirmed that the maximal effect of the novel compounds was weaker than that of rifampicin (Figure 7D). It should be noted that the decline in activation by 30 µM of compound **42**, as compared to 10 µM, might result from beginning cytotoxicity in H-P cells (see Figure S2). Comparison of the EC_50_ values of the compounds, which have been derived from the concentration response analyses, to rifampicin EC_50_ showed that **25** is less potent in PXR activation than rifampicin (Figure 7E). A similar trend was observed for **42** and **53**, however with *p* values of 0.1126 (paired t-test) and 0.1027, respectively, failed to reach statistical significance. Thirdly, we analyzed the induction of endogenous PXR target gene expression by compound treatment of differentiated HepaRG hepatocytes. Figure 7F shows that the prototypical PXR agonist rifampicin induced the expression of *ABCB1*, *CYP2B6*, and *CYP3A4*. In contrast, **25** and **53** demonstrated induction of *CYP2B6* and *CYP3A4* only, while they did not, or only very weakly, induce *ABCB1*. The extent of *CYP2B6* induction by the novel compounds was as high as by rifampicin, while induction of *CYP3A4* tended to be weaker.

The difference in *CYP2B6* and *CYP3A4* induction may indicate that the novel compounds may also act on the constitutive androstane receptor (CAR, *NR1I3*), of which *CYP2B6* is the prototypical target gene [81]. As vitamin D receptor (VDR, *NR1I1*) activation also results in induction of hepatic cytochrome P450 genes [82], we analyzed the specificity of the novel compounds with regard to the activation of these two receptors, which are the closest relatives of PXR (*NR1I2*). Neither of the compounds activated VDR per se nor interfered with vitamin D activation of the receptor (Figure S3). Regarding CAR, we analyzed effects on the two main hepatic isoforms [83], the constitutively active reference variant CAR1, and the ligand-dependent isoform CAR3. Of the three compounds, **25** and **53** demonstrated activation of CAR1, which in case of **53** was confirmed further by its ability to resolve inhibition of CAR1 by the inverse agonist CINPA1 (Figure S3). All three novel compounds demonstrated activation of CAR3, whereby **25** enhanced the activation by the prototypical CAR ligand CITCO even synergistically. In conclusion, the novel PXR agonists identified here also demonstrated activation of CAR, which might explain that they activated *CYP2B6* in hepatocytes more strongly than *CYP3A4*.

## 4. Conclusions

Due to the large size and flexibility of the ligand binding pocket of the PXR, the discrimination of activators and non-activators is a challenging task, also for machine learning approaches. In order to improve the performance of machine learning models in PXR activator prediction, we designed a new regularization technique that penalizes the performance gap between training and validation data during the model selection in hyperparameter tuning. We deployed this technique on RF models and SVMs using fingerprint as well as physicochemical properties as the underlying training features. Whereas the regularized models showed a comparable performance on the training data, their MCC values for the test set were up to 0.21 higher than those of the baseline models. In a prospective screening experiment with the regularized models, 12 of the 31 purchased and tested compounds were confirmed in a PXR-LBD assembly assay to exhibit an activation of more than 10% of rifampicin activity, thereby indicating ligand binding to PXR. Importantly, these compounds are structurally distinct from any known PXR ligands. Hit follow-up studies resulted in a number of bioactive compounds, of which we studied three representatives, **25**, **42**, and **53**, in detail, to further corroborate interaction with PXR and distinguish between agonists and antagonist properties, as the PXR-LBD assembly assay does not discriminate respectively. According to the combined results from (1) mammalian two-hybrid interaction assays with co-repressor and co-activator proteins, (2) PXR-dependent reporter gene assays and (3) chemical induction experiments in differentiated hepatocytes, the representative compounds proved to act as agonists for PXR. Future research is however required to confirm direct physical interaction, i.e., ligand binding, to the receptor using a biochemical assay.

In summary, we have demonstrated the successful development and application of machine learning models to identify novel PXR activators. The presented regularization technique is widely applicable and is expected to be particularly useful when modeling targets with complex activity landscapes or training on highly biased data sets.

## Figures and Tables

**Table 1:** Filter criteria and number of compounds removed during each step of the data preprocessing.

	PubChem PXR Data Set	ToxCast PXR Data Set	Literature PXR Data Set
Initial data set	2864	3626	434
Missing identifier (CID/CAS number) or SMILES	14	308	0
Inconclusive activity	488	776	0
MW ≤ 200 Da	1219	1339	16
Presence of inorganic elements	23	2	0
Contradicting class labels	24	5	0
Duplicates	155	17	9
Final PXR data set	941 (A: 202, N: 739) ^1^	1179 (A: 642, N: 537)	409 (A: 250, N: 159)

^1^ Numbers in parenthesis indicate the number of activators (A) and non-activators (N) in the final PXR data set.

**Table 2:** Overview of the Composition of PXR Data Sets Utilized in this Work.

	PubChem PXR Data Set	ToxCast PXR Data Set	Literature PXR Data Set
No. compounds	941	1179	409
No. scaffolds	387	470	259
No. drugs scaffolds	187 (4%) ^1^	224 (4%)	145 (3%)
No. cosmetics scaffolds	108 (15%)	154 (22%)	36 (5%)
No. pesticides scaffolds	170 (25%)	171 (25%)	51 (7%)

^1^ Numbers in parenthesis indicate the percentages of scaffolds with respect to the reference set.

**Table 3:** Performance of Different ML Models and Feature Sets. ^1^.

Model			Cross-Validation on the PubChem PXR Data Set		Testing on the			
					ToxCast PXR Data Set		Literature PXR Data Set	
ML Algorithm	Feature Set(s)	Gap-Penalization ^2^	MCC ^3^	AUC ^3^	MCC	AUC	MCC	AUC
RF	PC	no	0.43 (±0.04)	0.83 (±0.04)	0.41	0.82	0.10	0.59
		yes	0.43 (±0.05)	0.80 (±0.04)	0.46	0.81	0.21	0.57
	FP	no	0.45 (±0.07)	0.82 (±0.03)	0.25	0.75	0.09	0.55
		yes	0.31 (±0.04)	0.76 (±0.01)	0.28	0.72	0.02	0.55
	PC + FP	no	0.48 (±0.06)	0.83 (±0.03)	0.35	0.81	0.03	0.56
		**yes**	**0.42 (±0.06)**	**0.81 (±0.03)**	**0.47**	**0.82**	**0.24**	**0.59**
SVM	PC	no	0.41 (±0.06)	0.80 (±0.03)	0.40	0.77	0.14	0.56
		yes	0.41 (±0.06)	0.80 (±0.02)	0.44	0.81	0.13	0.56
	FP	no	0.46 (±0.07)	0.82 (±0.03)	0.31	0.77	0.00	0.54
		yes	0.32 (±0.06)	0.77 (±0.03)	0.34	0.74	0.06	0.52
	PC + FP	no	0.48 (±0.10)	0.86 (±0.03)	0.44	0.82	0.00	0.55
		yes	0.42 (±0.03)	0.81 (±0.02)	0.45	0.82	0.15	0.55

^1^ The model performing best on unseen data (RF classifier trained on physicochemical features and fingerprints, with gap-penalization) is indicated in bold. ^2^ Indicates whether the optimization score penalized the train-test performance gap. ^3^ Numbers in parentheses indicate standard deviations. The bold formatted text marks the overall best model.

**Table 4:** Overview of Compounds Selected by Virtual Screening.

ID	Hit Compounds	Nearest Neighbor in Training Set	Similarity of Hit Compound to Nearest Neighbor	Cluster ID	Measured Activity ^1^	S.D. ^2^
**1**	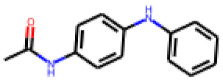 MolPort-001-946-370	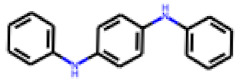 PubChem	0.57	A	0.15	1.09
**2**	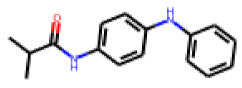 MolPort-009-220-213	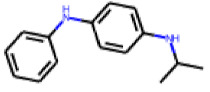 ToxCast	0.52	A	1.41	1.59
**3**	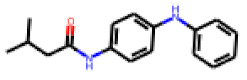 MolPort-003-820-268	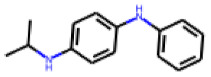 PubChem	0.47	A	9.41	4.72
**4**	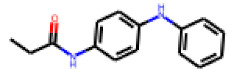 MolPort-001-823-879	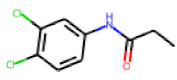 ToxCast	0.51	A	1.59	1.66
**5**	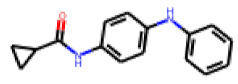 MolPort-001-529-219	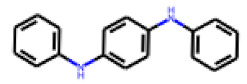 PubChem	0.48	A	1.10	0.02
**6**	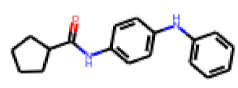 MolPort-001-545-599	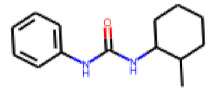 PubChem	0.45	A	2.78	0.80
**7**	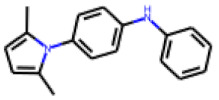 MolPort-000-993-714	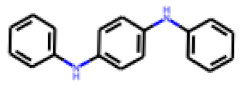 PubChem	0.54	A	8.65	7.57
**8**	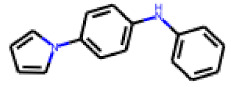 MolPort-000-993-856	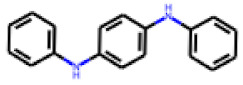 PubChem	0.59	A	4.25	1.97
**9**	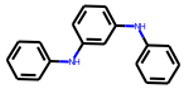 MolPort-001-838-155	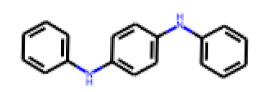 PubChem	0.71	A	32.77	13.01
**10**	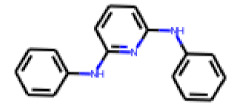 MolPort-002-238-843	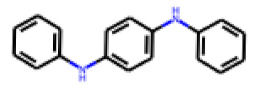 PubChem	0.52	A	5.88	0.38
**11**	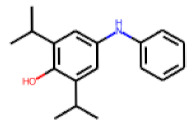 MolPort-000-279-714	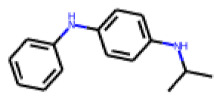 ToxCast	0.47	A	33.00	19.54
**12**	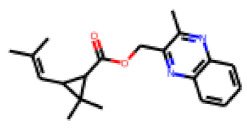 MolPort-020-102-538	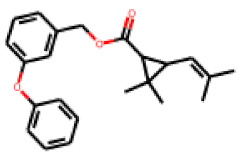 ToxCast	0.53	B	17.00	6.15
**13**	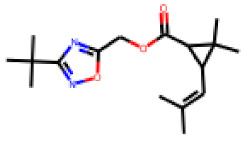 MolPort-027-674-395	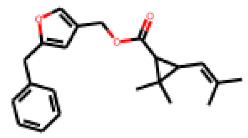 ToxCast	0.44	B	28.00	4.97
**14**	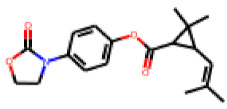 MolPort-027-691-387	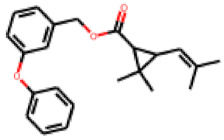 ToxCast	0.37	B	0.34	2.18
**15**	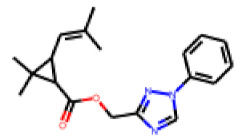 MolPort-027-933-109	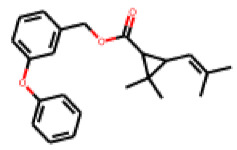 ToxCast	0.52	B	26.50	8.41
**16**	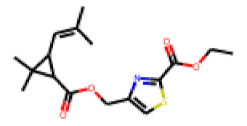 MolPort-035-741-775	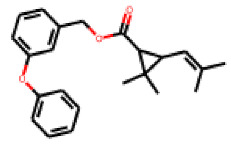 ToxCast	0.44	B	4.10	0.46
**17**	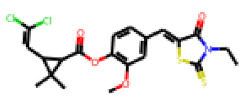 MolPort-003-881-027	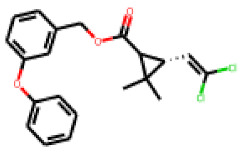 ToxCast	0.33	B	44.80	14.97
**18**	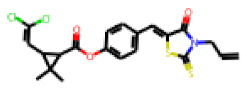 MolPort-003-881-070	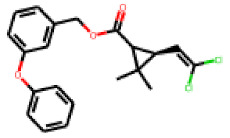 ToxCast	0.34	B	39.63	19.31
**19**	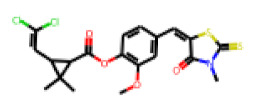 MolPort-003-881-625	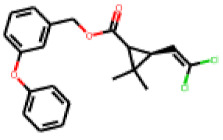 ToxCast	0.32	B	9.54	1.57
**20**	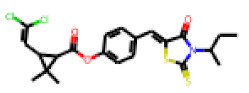 MolPort-002-237-349	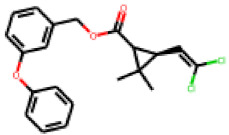 ToxCast	0.34	B	4.83	2.00
**21**	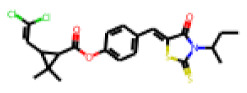 MolPort-000-196-136	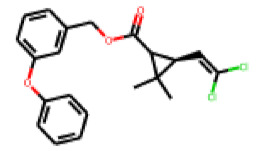 ToxCast	0.34	B	7.21	3.28
**22**	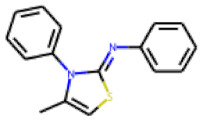 MolPort-000-431-925	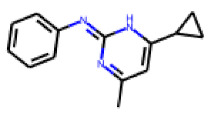 ToxCast	0.26	singleton	91.00	36.91
**23**	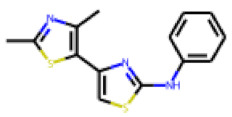 MolPort-000-690-666	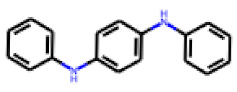 PubChem	0.30	singleton	11.08	5.40
**24**	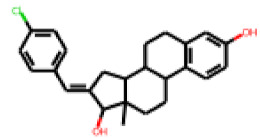 MolPort-005-280-909	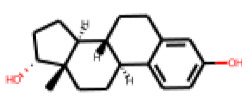 ToxCast	0.58	singleton	1.34	0.71
**25**	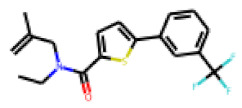 MolPort-006-630-224	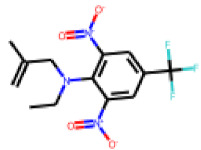 PubChem	0.36	singleton	136.87	28.01
**26**	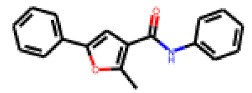 MolPort-001-619-443	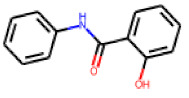 ToxCast	0.46	singleton	20.11	9.51
**27**	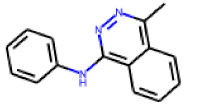 MolPort-002-547-842	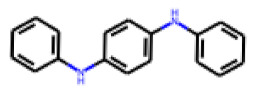 PubChem	0.39	singleton	10.14	1.55
**28**	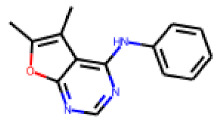 MolPort-002-656-531	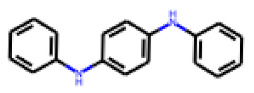 PubChem	0.31	singleton	0.34	0.63
**29**	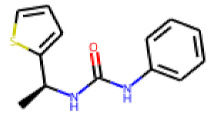 MolPort-001-991-071	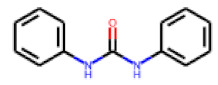 PubChem	0.47	singleton	4.49	1.11
**30**	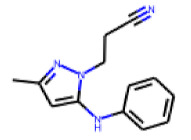 MolPort-004-004-399	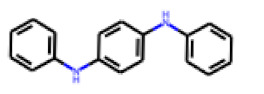 PubChem	0.29	singleton	0.02	1.81
**31**	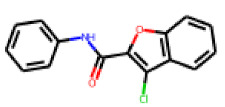 MolPort-007-570-291	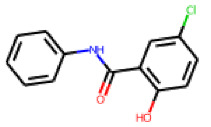 ToxCast	0.41	singleton	15.32	14.08

^1^ HepG2 cells were co-transfected with expression plasmids encoding GAL4-DBD/PXR-LBD (132-188) and VP16-AD/PXR-LBD (189–434) fusion proteins and GAL4-dependent firefly luciferase reporter gene plasmid pGL3-G5. Transfected cells were treated with 0.1% DMSO, 10 µM rifampicin or 10 µM of respective test compounds (**1** to **31**) for 24 h. Data are shown as means ± S.D. (*n* = 3) of % activation. Activity obtained by 10 µM rifampicin was set as 100%. ^2^ Standard deviation of the measurements.

**Table 5:** Compounds tested in follow-up experiments related to **25**.

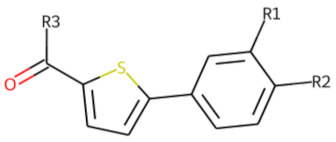					
ID	R1	R2	R3	Measured Activity ^1^	S.D. ^2^
**32** MolPort-006-630-016	CF_3_	H	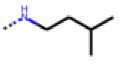	13.19	4.02
**33** MolPort-006-630-300	CF_3_	H	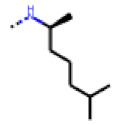	37.32	5.57
**34** MolPort-006-629-818	CF_3_	H	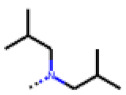	100.91	15.25
**35** MolPort-006-630-013	CF_3_	H	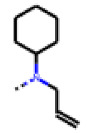	56.85	7.23
**36** MolPort-006-629-816	CF_3_	H	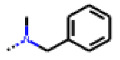	12.59	4.53
**37** MolPort-006-629-973	CF_3_	H	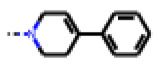	4.79	1.33
**38** MolPort-006-630-273	H	OCH_2_CH_3_	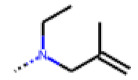	14.11	2.52
**39** MolPort-006-630-110	H	F	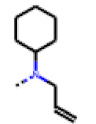	80.37	25.75
**40** MolPort-006-630-237	H	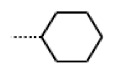	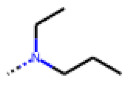	6.43	1.57

^1^ % activation of rifampicin activity, achieved by 10 µM of respective test compounds (32 to 40), was determined by PXR-LBD assembly assay in transfected HepG2 cells, as described in the legend of Table 4. ^2^ Standard deviation of the measurements.

**Table 6:** Compounds tested in follow-up experiments related to **22**.

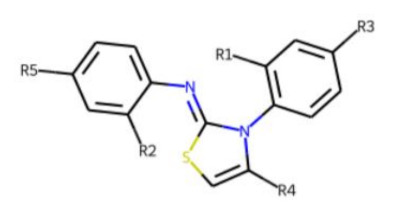							
ID	R1	R2	R3	R4	R5	Measured Activity ^1^	S.D. ^2^
**41** MolPort-003-179-500	H	H	H	COH	H	6.27	0.87
**42** MolPort-000-431-927	H	H	Cl	Me	Cl	115.84	17.76
**43** MolPort-000-431-934	H	H	Me	Me	Me	71.67	15.93
**44** * MolPort-020-176-525	Me	Me	H	Me	H	215.49	31.07
**45** MolPort-004-827-215	Me	Me	H	4-BrC6H4	H	9.25	4.38

^1^ % activation of rifampicin activity, achieved by 10 µM of respective test compounds (41 to 45), was determined by PXR-LBD assembly assay in transfected HepG2 cells, as described in the legend of Table 4. ^2^ Standard deviation of the measurements. * Note that during subsequent MS analyses **44**, the most promising compound related to **22**, did not meet the required purity threshold of ≥90%.

**Table 7:** Compounds tested in follow-up experiments related to **17**.

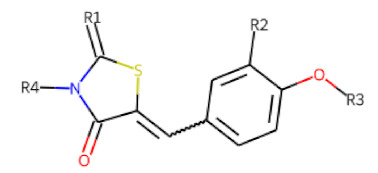							
ID	R1	R2	R3	R4	Configuration	Measured Activity ^1^	S.D. ^2^
**46** MolPort-002-173-405	S	H	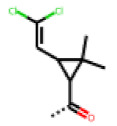	(CH_2_)_2_OCH_3_	E	12.45	5.06
**47** MolPort-003-881-006	S	H	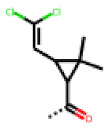	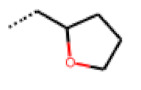	Z	21.96	2.87
**48** MolPort-003-881-001	S	H	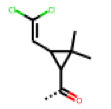	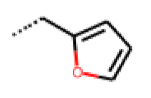	Z	19.72	4.24
**49** MolPort-000-419-900	S	H	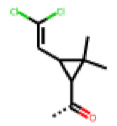	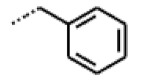	Z	8.54	2.04
**50** MolPort-003-880-748	S	OCH_3_	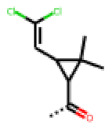	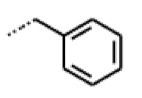	Z	6.66	1.30
**51** MolPort-003-881-514	NH	OCH_3_	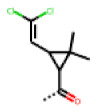	H	Z	4.10	1.63
**52** MolPort-044-415-081	S	OCH_3_	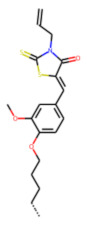	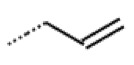	E/Z	15.73	4.05
**53** MolPort-002-216-386	S	OCH_3_	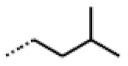	Prop	Z	84.18	23.30
**54** MolPort-001-899-455	S	OCH_3_	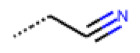	Et	E	19.46	8.00
**55** MolPort-001-552-801	S	OCH_3_	Et	Et	Z	18.65	6.09
**56** MolPort-039-019-225	S	OCH_2_CH_3_	Prop	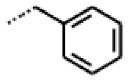	Z	22.63	10.48
**57** * MolPort-001-634-394	S	OCH_2_CH_3_	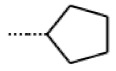	Et	Z	95.59	45.14

^1^ % activation of rifampicin activity, achieved by 10 µM of respective test compounds (**46** to **57**), was determined by PXR-LBD assembly assay in transfected HepG2 cells, as described in the legend of Table 4. ^2^ Standard deviation of the measurements. * Note that during subsequent MS analyses **57**, the most promising compound related to **17**, did not meet the required purity threshold of ≥90%.

## Data Availability

All data sets used in this study for machine learning are publicly available. All experimental data are reported in the figures in the manuscript and as supplementary materials.

## Supplementary Materials

The following supporting information can be downloaded at: https://www.mdpi.com/article/10.3390/cells11081253/s1, Table S1: Combinations of Hyperparameters Explored by Grid Search; Table S2: Optimal Parameters for Each Random Forest Model; Table S3: Optimal Parameters for Each Support Vector Machine; Table S4: Important Features in the Random Forest Model Trained on Physicochemical and Fingerprint Features, and Optimized with Gap Penalization; Figure S1: Mass-spec data; Figure S2: Cell toxicity of novel compounds. Figure S3: Selectivity of novel compounds within the NR1I group of nuclear receptors.
