# Supplementary material for: Development and Experimental Validation of Regularized Machine Learning Models Detecting New, Structurally Distinct Activators of PXR
Source: Cells. 2022 Apr 7;11(8):1253. doi: 10.3390/cells11081253 (PMC9029776; doi:10.3390/cells11081253)
Supplement: Supplementary file 1 [file cells-11-01253-s001.zip › cells-1639087-supplementary.pdf]

Supplementary Information

Development and experimental validation of regularized machine learning models detecting new, structurally distinct activators of PXR

Steffen Hirte [1], Oliver Burk [2,3], Ammar Tahir [4], Matthias Schwab [2,5,6], Björn Windshügel [7,8], Johannes Kirchmair [1,*]

[1] Department of Pharmaceutical Sciences, Division of Pharmaceutical Chemistry, Faculty of Life Sciences, University of Vienna, 1090 Vienna, Austria; steffen.hirte@univie.ac.at, johannes.kirchmair@univie.ac.at

[2] Dr. Margarete Fischer-Bosch-Institute of Clinical Pharmacology, Stuttgart; Oliver.Burk@ikp-stuttgart.de

[3] University of Tübingen, Tübingen, Germany

[4] Department of Pharmaceutical Sciences, Division of Pharmacognosy, Faculty of Life Sciences, University of Vienna, 1090 Vienna, Austria; ammar.tahir@univie.ac.at

[5] Departments of Clinical Pharmacology and Biochemistry and Pharmacy, University of Tuebingen, Tübingen, Germany; matthias.schwab@med.uni-tuebingen.de

[6] Cluster of Excellence IFIT (EXC 2180) "Image-Guided and Functionally Instructed Tumor Therapies", University of Tübingen, Tübingen, Germany

[7] Fraunhofer Institute for Translational Medicine and Pharmacology ITMP, Discovery Research ScreeningPort, Hamburg, Germany; Bjoern.Windshuegel@itmp.fraunhofer.de

[8] Department of Life Sciences and Chemistry, Jacobs University Bremen, Bremen, Germany

* Correspondence: johannes.kirchmair@unvie.ac.at; +43-1-4277-55104

Table S1. Combinations of Hyperparameters Explored by Grid Search.

Technique	Parameters	Explored values
RandomForestClassifier	n_estimators	500
	min_samples_split	0, 1, 2, 4, 8, 16, 32, 64, 128, 256, 512
	min_samples_leaf	0, 1, 2, 4, 8, 16, 32, 64, 128, 256, 512
	max_features	log2, sqrt, 0.1, 0.2, 0.3, 0.4, 0.5
	class_weight	Balanced / None
SVC	Min-Max-Scaler	Yes / No
	C	10^{-2}, 10^{-1}, 10^{0}, 10^{1}, 10^{2}, 10^{3}, 10^{4}, 10^{5}
	gamma	10^{-9}, 10^{-8}, 10^{-7}, 10^{-6}, 10^{-5}, 10^{-4}, 10^{-3}, 10^{-2}, 10^{-1}
	class_weight	Balanced / None

Table S2. Optimal Parameters for Each Random Forest Model.

Features	Gap penalization	min_samples_split	min_samples_leaf	max_features	class_weight
PC	no	16	1	0.3	balanced
	yes	128	16	0.1	balanced
FP	no	2	2	log2	balanced
	yes	256	16	log2	balanced
PC + FP	no	64	2	log2	balanced
	yes	256	32	0.1	balanced

Table S3. Optimal Parameters for Each Support Vector Machine.

Features	Gap penalization	MinMaxScaler	C	gamma	class_weight
PC	no	no	1000	10^{-4}	balanced
	yes	no	100	10^{-5}	balanced
FP	no	no	10000	10^{-6}	balanced
	yes	no	1	10^{-3}	balanced
PC + FP	no	no	1000	10^{-5}	balanced
	yes	no	100	10^{-5}	balanced

Table S4. Important Features in the Random Forest Model Trained on Physicochemical and Fingerprint Features, and Optimized with Gap Penalization.

Rank	Feature	Importance	Rank	Feature	Importance
1	esol	0.162183	43	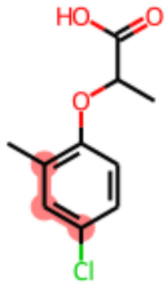	0.001799
2	refractivity	0.121561	44	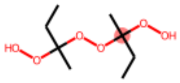	0.001656
3	logp	0.118305	45	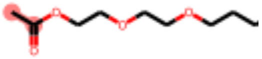	0.001443
4	n_heavy	0.069898	46	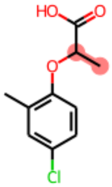	0.001356
5	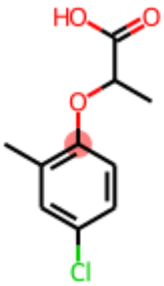	0.057798	47	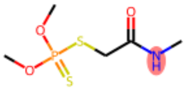	0.001290

6	tpsa	0.050792	48	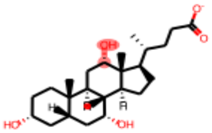	0.001228
7	n_rings	0.043200	49	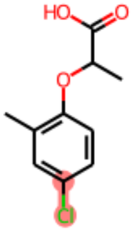	0.001209
8	weight	0.040083	50	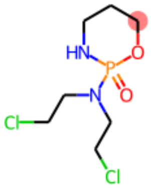	0.001094
9	frac_rotable_bonds	0.028083	51	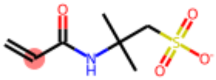	0.001087
10	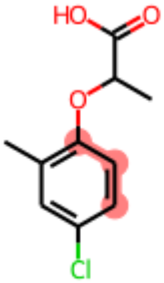	0.026525	52	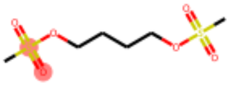	0.000996
11	n_ar	0.021032	53	n_halogens	0.000941
12	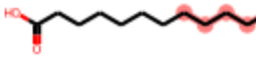	0.018964	54	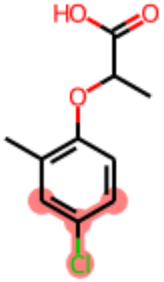	0.000938

13	n_rotatable_bonds	0.018753	55	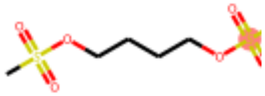	0.000921
14	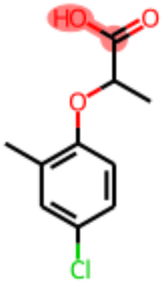	0.018139	56	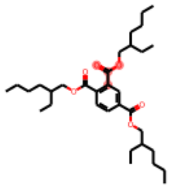	0.000804
15	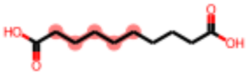	0.018040	57	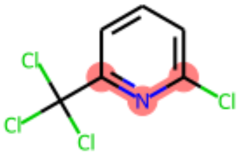	0.000758
16	n_sp3c	0.016106	58	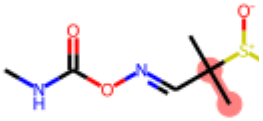	0.000737
17	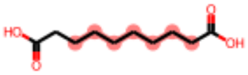	0.013996	59	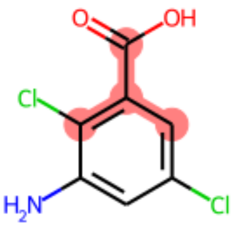	0.000696

18	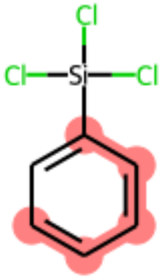	0.012989	60	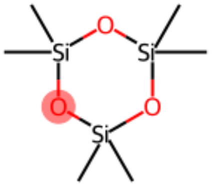	0.000681
19	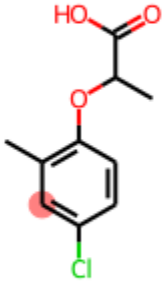	0.010226	61	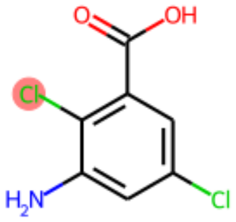	0.000594
20	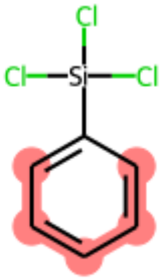	0.009552	62	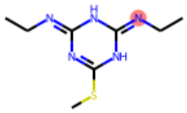	0.000573
21	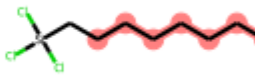	0.009350	63	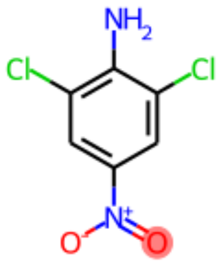	0.000527
22	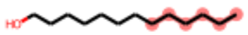	0.008720	64	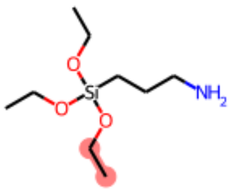	0.000522

23	n_O	0.007863	65	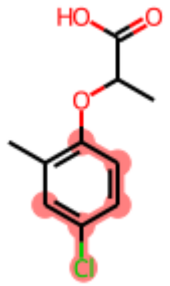	0.000422
24	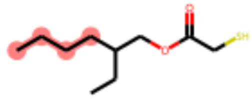	0.006749	66	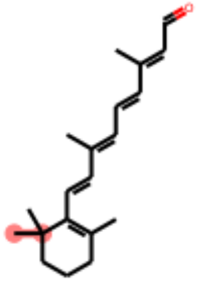	0.000391
25	n_hbd	0.006624	67	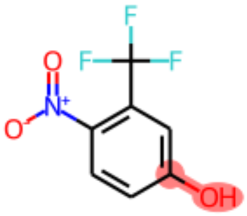	0.000387
26	n_hba	0.005833	68	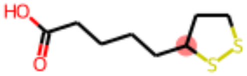	0.000380
27	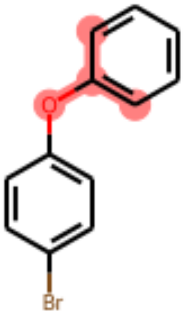	0.004984	69	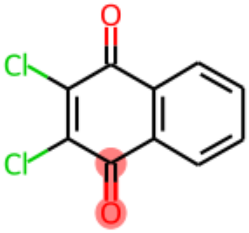	0.000347

28	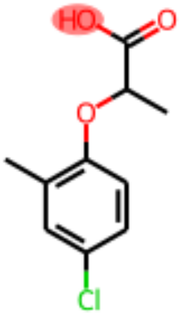	0.004887	70	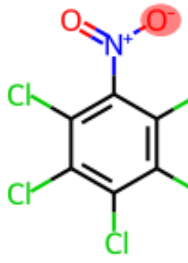	0.000321
29	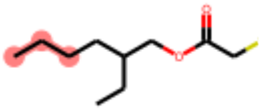	0.004461	71	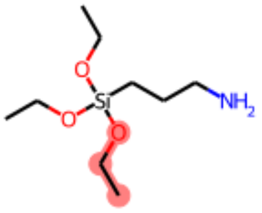	0.000249
30	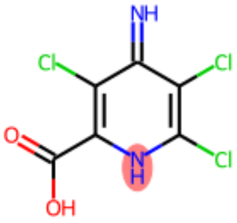	0.004232	72	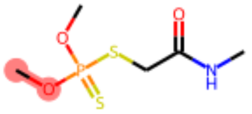	0.000225
31	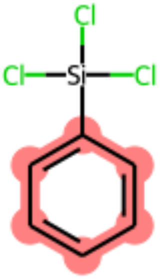	0.004222	73	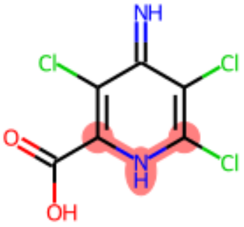	0.000194
32	n_N	0.003953	74	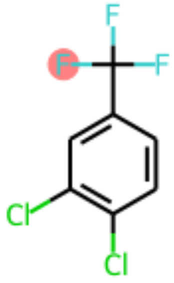	0.000190

33	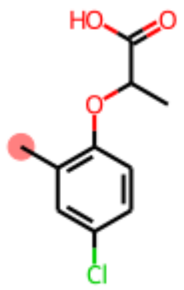	0.003533	75	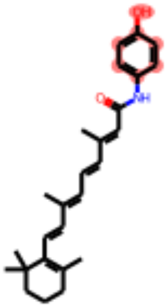	0.000189
34	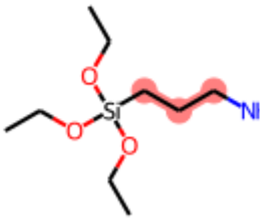	0.003365	76	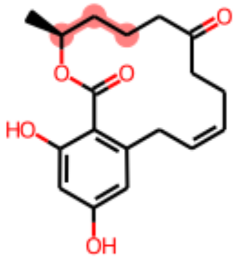	0.000163
35	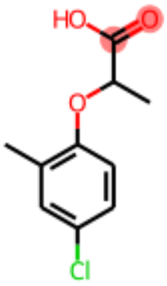	0.003153	77	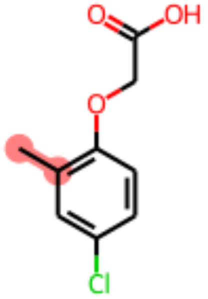	0.000149
36	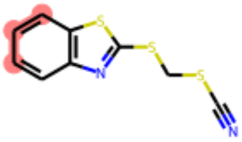	0.002655	78	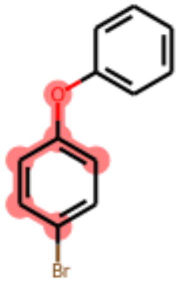	0.000136
37	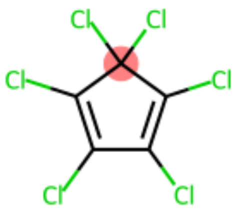	0.002603	79	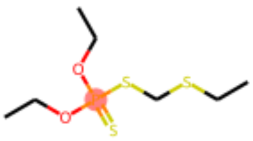	0.000128

38	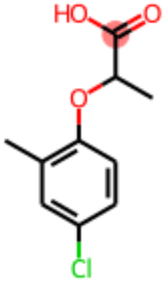	0.002485	80	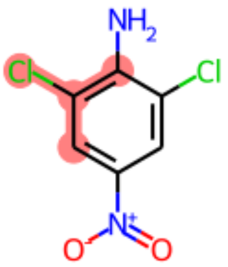	0.000069
39	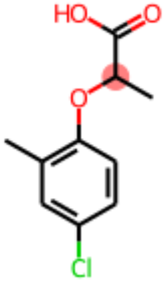	0.002194	81	n_S	0.000053
40	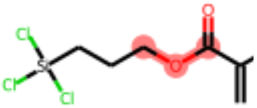	0.002129	82	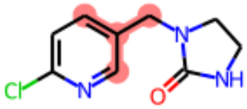	0.000044
41	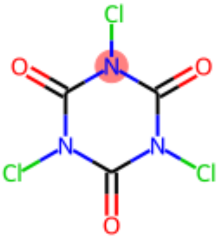	0.001955	83	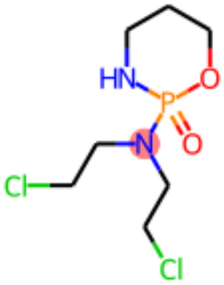	0.000036
42	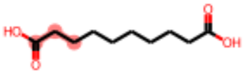	0.001875	84	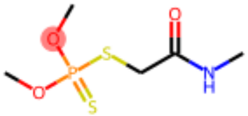	0.000025

[1] Most frequent pattern illustrated in an example molecule and highlighted in red.

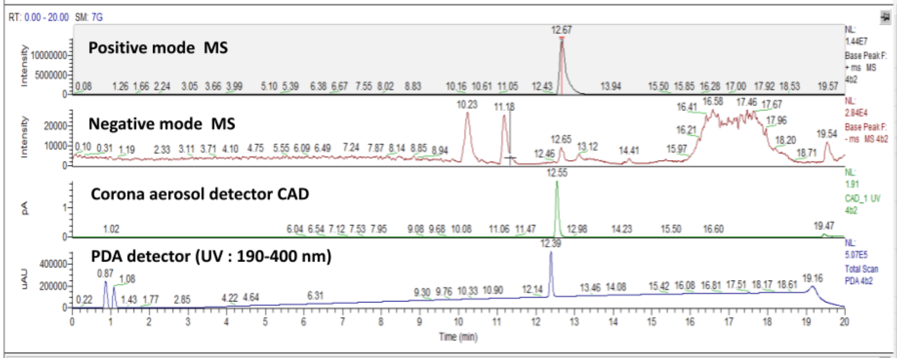

PEAK LIST						
4b2.raw						
RT: 0.00 - 19.99						
Number of detected peaks: 34						
Apex RT	Start RT	End RT	Area	%Area	Height	%Height
1.02	0.94	1.13	0.1	1.06	0.02	1.01
1.24	1.17	1.36	0.016	0.17	0.003	0.13
7.95	7.92	7.98	0.001	0.02	0.001	0.04
8	7.98	8.04	0.002	0.02	0.001	0.04
8.74	8.71	8.78	0.003	0.03	0.001	0.06
9.01	8.97	9.03	0.002	0.02	0.001	0.04
9.08	9.03	9.13	0.006	0.06	0.002	0.08
9.17	9.13	9.21	0.002	0.03	0.001	0.05
9.35	9.28	9.37	0.005	0.05	0.002	0.08
9.48	9.41	9.53	0.004	0.05	0.001	0.05
9.6	9.58	9.65	0.002	0.03	0.001	0.05
9.68	9.67	9.71	0.002	0.02	0.001	0.06
10.08	9.97	10.17	0.052	0.55	0.008	0.4
10.22	10.2	10.26	0.004	0.04	0.001	0.06
10.28	10.26	10.29	0.001	0.02	0.001	0.05
10.31	10.29	10.34	0.003	0.03	0.001	0.06
10.45	10.39	10.51	0.021	0.22	0.004	0.19
10.53	10.51	10.59	0.008	0.09	0.003	0.17
10.72	10.62	10.75	0.008	0.08	0.001	0.07
11.06	10.98	11.17	0.04	0.43	0.007	0.36
11.34	11.29	11.43	0.007	0.07	0.001	0.06
12.55	12.47	12.64	8.552	90.33	1.808	90.73
13	12.95	13.05	0.004	0.04	0.001	0.05
13.52	13.5	13.55	0.002	0.02	0.002	0.08
13.69	13.6	13.74	0.003	0.04	0.001	0.04
14.22	14.16	14.31	0.008	0.09	0.002	0.08
15.39	15.36	15.42	0.003	0.03	0.001	0.04
15.5	15.42	15.58	0.035	0.37	0.008	0.39
16.6	16.53	16.69	0.011	0.12	0.002	0.11
16.92	16.81	17.05	0.008	0.08	0.001	0.04
19.47	19.39	19.58	0.489	5.17	0.091	4.55
19.72	19.67	19.77	0.014	0.15	0.004	0.18
19.78	19.77	19.82	0.008	0.08	0.004	0.21
19.89	19.83	19.98	0.04	0.42	0.008	0.4

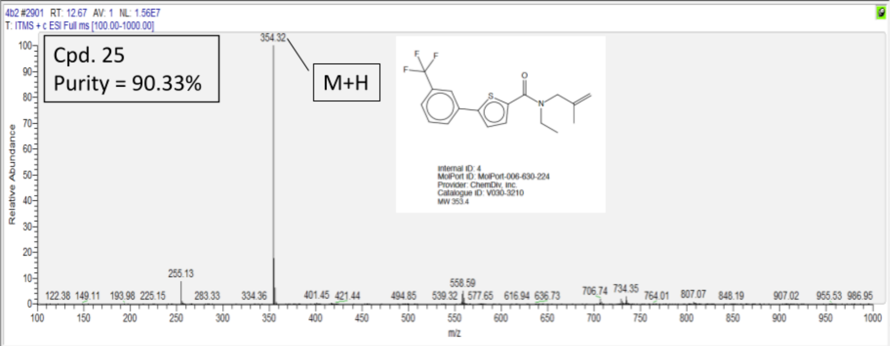

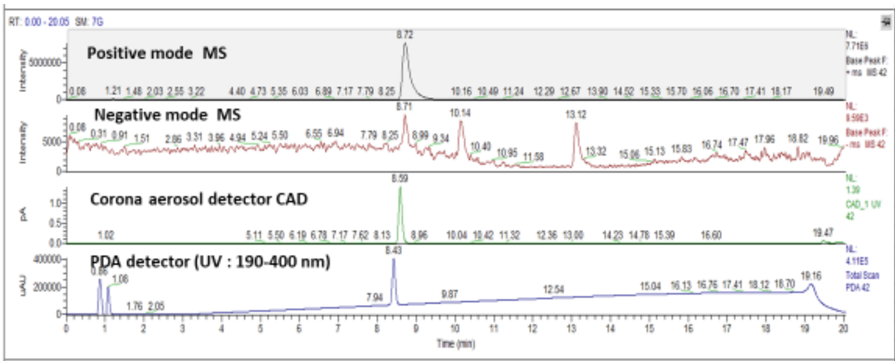

PEAK LIST						
42.raw						
RT: 0.00 - 20.00						
Number of detected peaks: 30						
Apex RT	Start RT	End RT	Area	%Area	Height	%Height
1.02	0.95	1.13	0.03	0.41	0.006	0.42
1.27	1.17	1.37	0.016	0.23	0.002	0.16
7.17	7.13	7.22	0.002	0.02	0	0.04
7.62	7.56	7.65	0.002	0.03	0.001	0.04
8.09	8.02	8.24	0.018	0.25	0.002	0.15
8.27	8.24	8.33	0.003	0.04	0.001	0.06
8.59	8.51	8.69	6.728	92.71	1.282	92.19
8.91	8.86	8.92	0.001	0.02	0.001	0.05
8.96	8.95	8.98	0.001	0.01	0.001	0.05
9.06	9.02	9.08	0.003	0.04	0.001	0.09
9.11	9.09	9.13	0.001	0.01	0.001	0.04
9.15	9.13	9.17	0.001	0.01	0.001	0.06
9.25	9.18	9.3	0.007	0.09	0.001	0.11
9.34	9.3	9.35	0.003	0.05	0.001	0.1
9.38	9.35	9.44	0.005	0.07	0.001	0.1
9.69	9.58	9.74	0.01	0.14	0.002	0.13
9.77	9.74	9.86	0.005	0.06	0.001	0.06
10.04	9.96	10.14	0.015	0.21	0.003	0.19
10.42	10.38	10.57	0.003	0.05	0	0.03
11.02	10.98	11.05	0.001	0.02	0.001	0.04
11.32	11.25	11.35	0.002	0.03	0.001	0.05
11.66	11.65	11.69	0.001	0.01	0.001	0.04
12.36	12.31	12.39	0.002	0.03	0.001	0.04
15.39	15.35	15.47	0.005	0.07	0.001	0.09
16.6	16.53	16.71	0.009	0.12	0.001	0.1
19.47	19.4	19.57	0.316	4.36	0.059	4.27
19.59	19.58	19.62	0.006	0.08	0.005	0.36
19.64	19.63	19.68	0.008	0.1	0.004	0.31
19.7	19.68	19.71	0.003	0.04	0.003	0.18
19.83	19.72	19.96	0.05	0.69	0.006	0.46

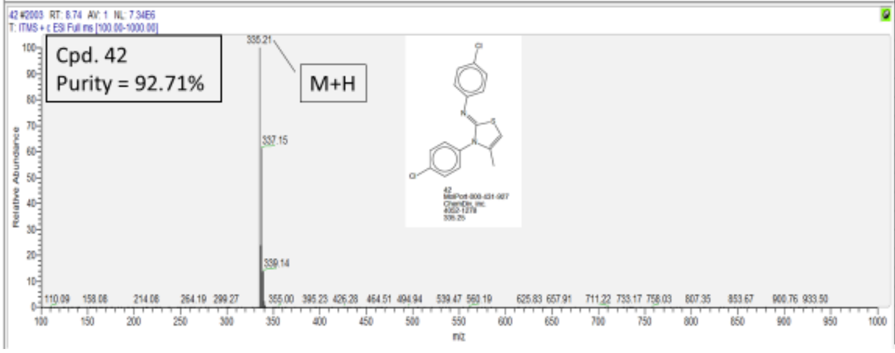

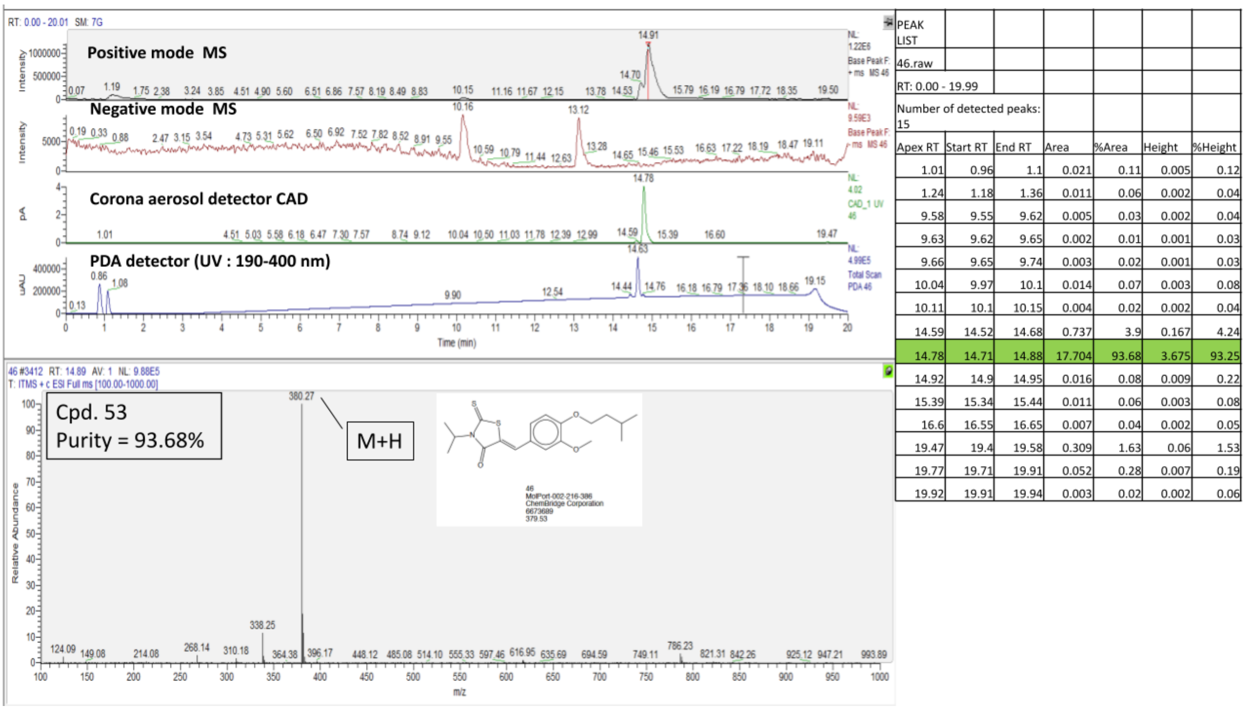

PEAK LIST						
46.raw						
RT: 0.00 - 19.99						
Number of detected peaks: 15						
Apex RT	Start RT	End RT	Area	%Area	Height	%Height
1.01	0.96	1.1	0.021	0.11	0.005	0.12
1.24	1.18	1.36	0.011	0.06	0.002	0.04
9.58	9.55	9.62	0.005	0.03	0.002	0.04
9.63	9.62	9.65	0.002	0.01	0.001	0.03
9.66	9.65	9.74	0.003	0.02	0.001	0.03
10.04	9.97	10.1	0.014	0.07	0.003	0.08
10.11	10.1	10.15	0.004	0.02	0.002	0.04
14.59	14.52	14.68	0.737	3.9	0.167	4.24
14.78	14.71	14.88	17.704	93.68	3.675	93.25
14.92	14.9	14.95	0.016	0.08	0.009	0.22
15.39	15.34	15.44	0.011	0.06	0.003	0.08
16.6	16.55	16.65	0.007	0.04	0.002	0.05
19.47	19.4	19.58	0.309	1.63	0.06	1.53
19.77	19.71	19.91	0.052	0.28	0.007	0.19
19.92	19.91	19.94	0.003	0.02	0.002	0.06

Figure S1. Mass-spec data.

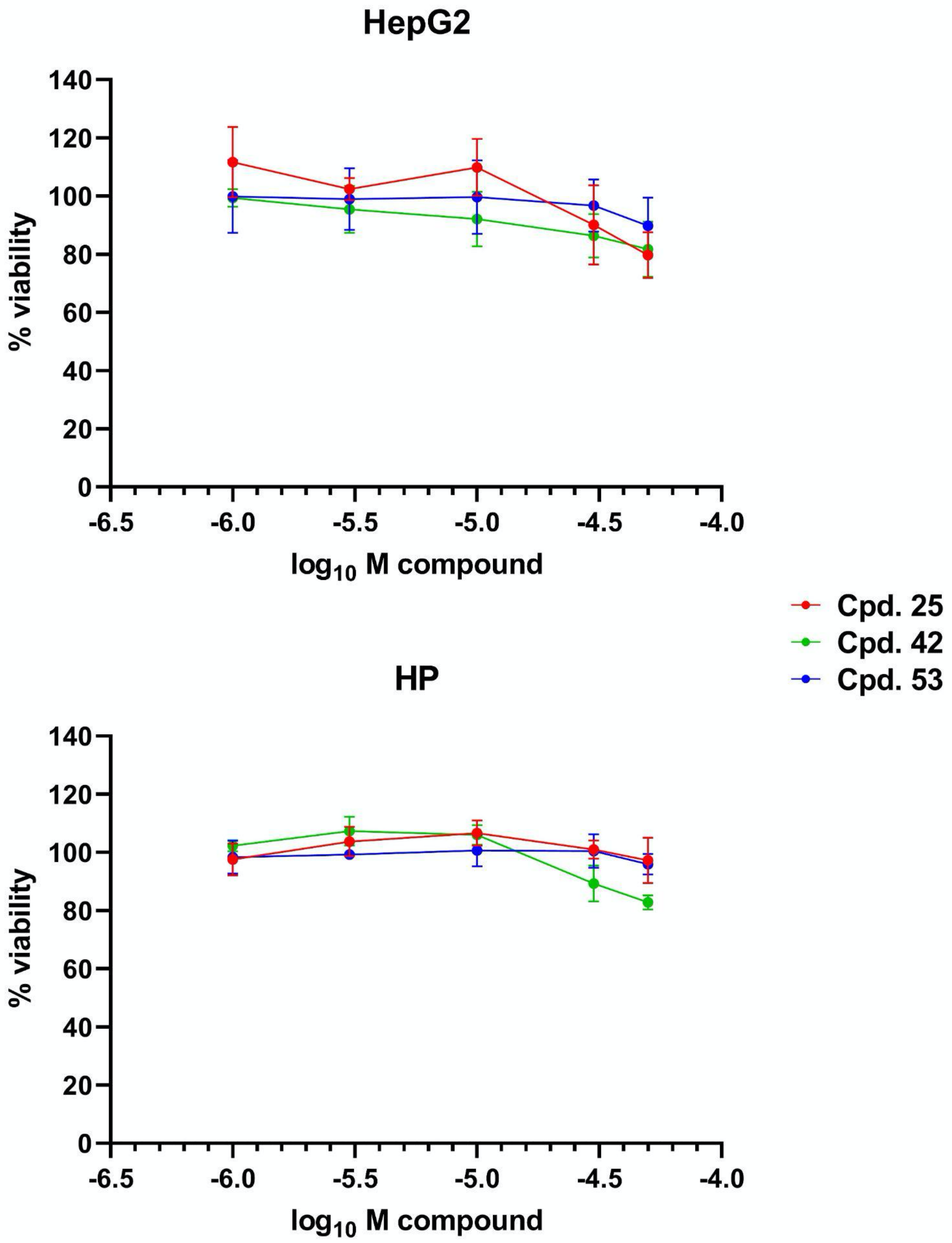

Figure S2. Cell toxicity of novel compounds. HepG2 or HP cells were treated with increasing concentrations (1-50 µM) of the indicated compounds for 24 h. Viability was determined based on ATP content and compared to the ATP content of cells treated with vehicle DMSO only (0.1% in case of compounds **25** and **42**, 0.17% for compound **53**), which was set as 100%, respectively. Means ± S.D. (n = 3) are shown.

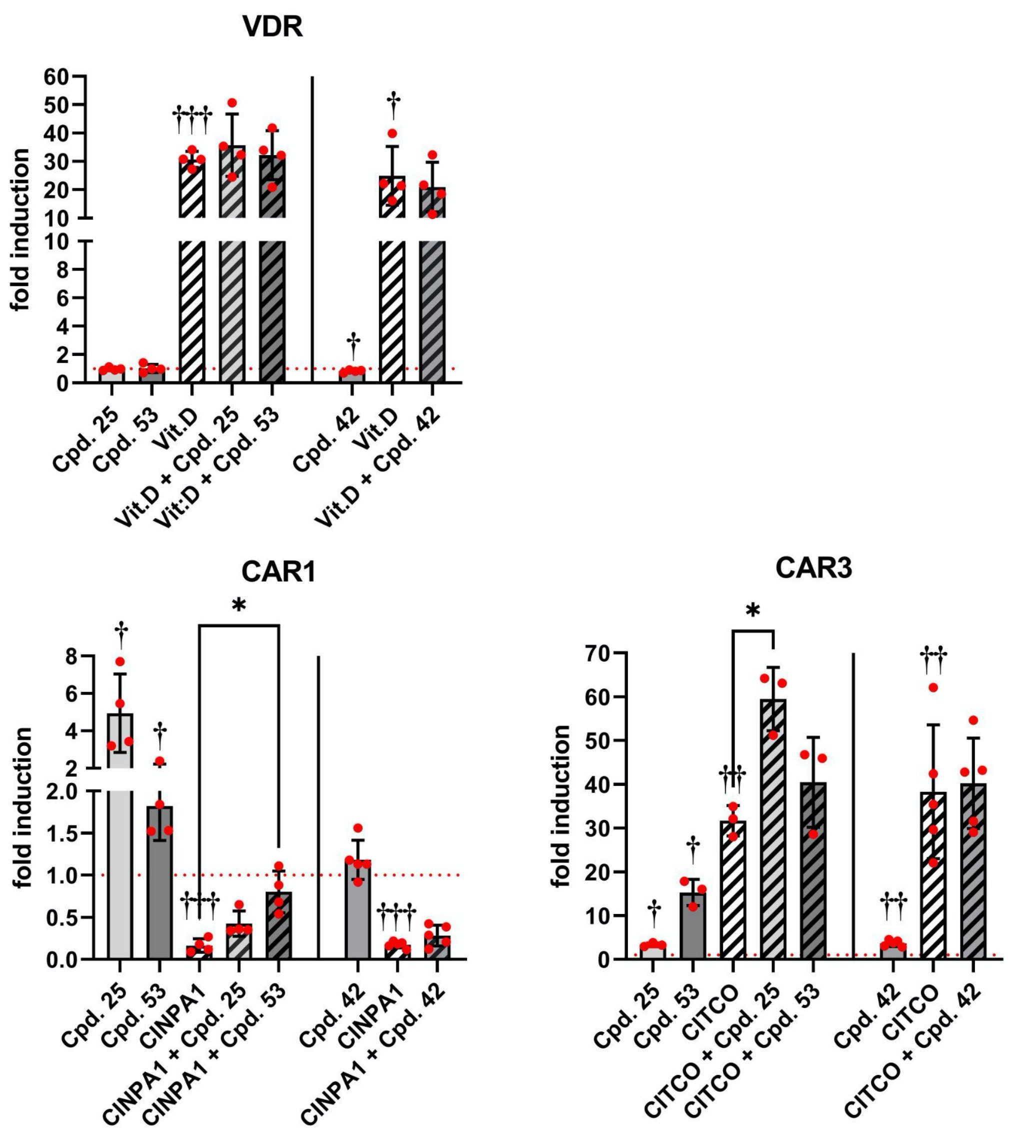

Figure S3. Selectivity of novel compounds within the NR1I group of nuclear receptors. HepG2 cells were transfected with expression plasmids encoding the indicated human nuclear receptors and treated for 24 h with 0.1 µM 1α,25-dihydroxyvitamin D3 (Vit.D), 1 µM CINPA1, 10 µM CITCO and/or 10 µM of the indicated compounds. In case of CAR3, RXRα expression plasmid was transfected additionally. Data are shown as means ± S.D. of normalized firefly luciferase activity of co—transfected reporter gene plasmids $(DR3)_3$-Tk (VDR) or CYP2B6 enhancer/promoter (CAR1, CAR3), relative to the activity of respectively transfected cells treated with 0.2% DMSO only, which was set as 1 (red dotted lines). The results of independent experiments (n ≥ 3) are illustrated as dots. Differences to respective treatments with DMSO only (daggers, exclusively for single compound treatments) were analyzed by one sample t-test. Differences to treatment with respective prototypical agonists (Vit.D – VDR; CITCO – CAR3) or inverse agonist (CINPA1 – CAR1) (asterisks, exclusively for co-treatments) were analyzed by repeated measures one-way ANOVA with Dunnett's multiple comparisons test. In the case of comparisons with co-treatments involving compound 42, paired t-test was applied instead. *, † p<0.05; †† p<0.01; ††† p<0.001.
